# Lapita Diet in Remote Oceania: New Stable Isotope Evidence from the 3000-Year-Old Teouma Site, Efate Island, Vanuatu

**DOI:** 10.1371/journal.pone.0090376

**Published:** 2014-03-05

**Authors:** Rebecca Kinaston, Hallie Buckley, Frederique Valentin, Stuart Bedford, Matthew Spriggs, Stuart Hawkins, Estelle Herrscher

**Affiliations:** 1 Department of Anatomy, University of Otago, Dunedin, New Zealand; 2 CNRS, Maison de l'Archéologie et de l'Ethnologie, Nanterre, France; 3 Department of Archaeology and Natural History, College of Asia and the Pacific, The Australian National University, Canberra, ACT, Australia; 4 School of Archaeology and Anthropology, The Australian National University, Canberra, ACT, Australia; 5 CNRS, Laboratoire Méditerranéen de Préhistoire Europe Afrique, Aix-Marseille Université, Aix-en-Provence, France; Ohio State University, United States of America

## Abstract

Remote Oceania was colonized ca. 3000 BP by populations associated with the Lapita Cultural Complex, marking a major event in the prehistoric settlement of the Pacific Islands. Although over 250 Lapita sites have been found throughout the Western Pacific, human remains associated with Lapita period sites are rare. The site of Teouma, on Efate Island, Vanuatu has yielded the largest burial assemblage (n = 68 inhumations) of Lapita period humans ever discovered, providing a unique opportunity for assessing human adaptation to the environment in a colonizing population. Stable isotope ratios (δ^13^C, δ^15^N, δ^34^S) of human bone collagen from forty-nine Teouma adults were analyzed against a comprehensive dietary baseline to assess the paleodiet of some of Vanuatu's earliest inhabitants. The isotopic dietary baseline included both modern plants and animals (n = 98) and prehistoric fauna from the site (n = 71). The human stable isotope data showed that dietary protein at Teouma included a mixture of reef fish and inshore organisms and a variety of higher trophic marine (e.g. marine turtle) and terrestrial animals (e.g. domestic animals and fruit bats). The domestic pigs and chickens at Teouma primarily ate food from a C_3_ terrestrial environment but their δ^15^N values indicated that they were eating foods from higher trophic levels than those of plants, such as insects or human fecal matter, suggesting that animal husbandry at the site may have included free range methods. The dietary interpretations for the humans suggest that broad-spectrum foraging and the consumption of domestic animals were the most important methods for procuring dietary protein at the site. Males displayed significantly higher δ^15^N values compared with females, possibly suggesting dietary differences associated with labor specialization or socio-cultural practices relating to food distribution.

## Introduction

**Figure 1:**
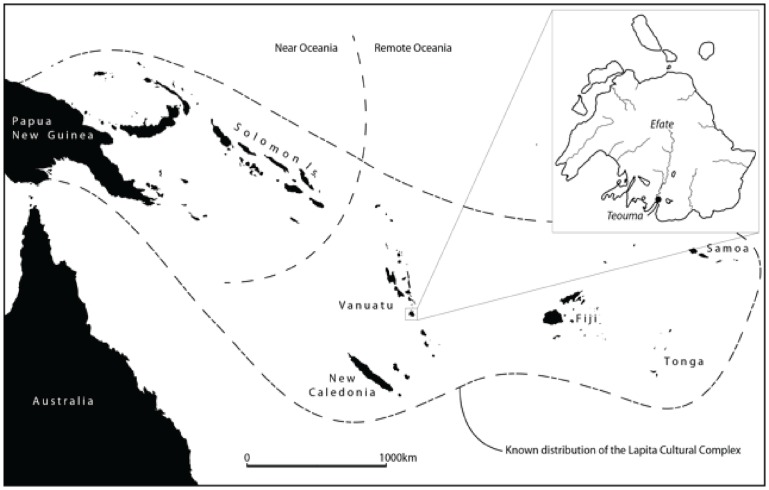
Map of Near and Remote Oceania and location of Efate Island, Vanuatu.

A change in diet and mode of subsistence can affect the health, demography and overall success of a population. The most well known example of a prehistoric dietary transition was the Agricultural Revolution that occurred in numerous centers across the globe, during which humans became increasingly reliant on domesticated plants and animals for food [1]–[3]. In the western Pacific Islands several major events can be viewed as catalysts for dietary transitions during prehistory after the initial arrival of humans in the region ca. 50,000 years ago. One significant event was the development of horticultural systems ca. 10,000 BP in the highlands of New Guinea [4]–[6]. Another occurred around 3300–3000 BP, when populations with cultural and biological links to Island South East Asia (ISEA), known as Lapita, appeared in the Bismarck Archipelago and then sailed into Remote Oceania with an established suite of domesticated plants and animals, a ‘transported landscape’, which enabled the settlement of previously uninhabited Pacific islands east of the Solomon Islands chain [7]–[9] (fig. 1).

As the Lapita populations sailed eastward and southward from the Bismarck Archipelago they left evidence of their occupation in the form of intricately decorated dentate-stamped Lapita pottery [8], [10], [11]. The numerous archaeological sites containing Lapita pottery in the Reef and Santa Cruz Islands, Vanuatu, New Caledonia, Fiji, Tonga, and Samoa attest to the success of Lapita colonization in Remote Oceania [7]. However, the marked decrease in biodiversity as one moves eastward across the Pacific Ocean required the initial Lapita settlers to adapt rapidly to the local ecology of each island to obtain enough food to support their fledgling communities [12]–[14].

A number of theories regarding the specific subsistence strategies of Lapita populations have been posited over the years. In the 1970s Groube [15] suggested that Lapita populations were Oceanic strandloopers focused primarily on gathering reef and inshore resources. Green [16] introduced the idea that domestic plants and animals transported with Lapita populations were the critical food items that facilitated the settlement of Remote Oceania [7], [17]. Other hypotheses regarding Lapita subsistence have also emerged arguing for a low level of plant cultivation and animal husbandry accompanied by the broad-spectrum exploitation of nearby marine and terrestrial resources [18], [19].

The presence of microfossils from exotic domestic plant species in Lapita and immediately post-Lapita sites in Remote Oceania indicates that horticulture was practiced during the early settlement of the region [20]–[24]. However, the extent to which the Lapita people (and their domestic animals) relied on this transported landscape compared with native flora and fauna during the initial settlement period remains uncertain [25]. A direct analysis of the diets of people from Lapita settlements and the dietary resources available to them would be ideal for addressing hypotheses regarding early Pacific island subsistence. The Teouma Lapita site, located on Efate Island, Vanuatu, provides this opportunity due to the large cemetery sample and the substantial amount of faunal material available for analysis from the site. Previous stable isotope analyses of Lapita-associated human skeletal remains has illuminated some aspects of Lapita diet [25]–[30], but the small nature of these samples and lack of site-specific baseline data has limited the scope of these interpretations.

The overall aim of this study is to assess human dietary adaptation in a recently colonized, pristine environment during the earliest known Lapita settlement of Central Vanuatu, on the far western edge of Remote Oceania. To achieve this we have analyzed the carbon, nitrogen and sulfur stable isotope ratios of human bone collagen to examine the diet of individuals interred in the Lapita cemetery of Teouma (ca. 3000–2900 BP) [31]. This study builds on a previous paleodietary analysis of thirty-three individuals interred in the Teouma cemetery conducted by Valentin et al. [32] by analyzing a further sixteen individuals and using sulfur stable isotope ratios to provide additional dietary information. To ensure the most reliable interpretation of the human stable isotope data we have analyzed modern plant and animal samples from Vanuatu in addition to prehistoric faunal samples from the Teouma site in order to establish the range of isotopic variation in the local environment. The prehistoric faunal remains include native and domestic species from Lapita (∼3000–2900 BP), late Lapita (2900–2800) and immediately post-Lapita (Arapus and Erueti) (∼2800–2500 BP) assemblages. The analysis of prehistoric faunal material from different temporal periods can also illuminate variation in animal diets that may be related to environmental change or animal husbandry techniques connected with certain activities such as horticultural intensification over time.

Stable isotope analysis has also been used to help discern whether there were dietary differences within a prehistoric cemetery sample that may be related to sex and age [33]–[35]. Differential access to certain foods within a population can provide information about broader socio-cultural factors in a prehistoric population, for example gender inequalities, labor specialization, immigration, social structure, and the treatment of certain members of a community, such as children [36]–[40]. Throughout the Pacific Islands certain foods are considered ‘high-status’ and may be reserved for highly esteemed individuals or simply eaten in larger quantities by these people [41]–[44]. These ‘high-status’ foods include fatty, greasy and oily animal products such as pig (*Sus scrofa*), chicken (*Gallus gallus*), marine turtle and some types of fish, especially pelagic species [45]–[47]. Linguistic reconstructions have given support to the theory that Lapita social structure was ranked and formed the basis of the highly stratified societies that developed later in Polynesia [7], [48]–[51]. Both within Polynesia and the Western Pacific, highly ranked individuals such as chiefs are traditionally male and these men usually have preferential access to high-status foods [44].

In this study we have tested two hypotheses in an attempt to address questions regarding Lapita subsistence strategies and possible differences in food consumption patterns related to sex. As people interred in the Teouma Lapita cemetery are thought to be some of the earliest colonists of Vanuatu [31] we hypothesize that the individuals from Teouma would have been more reliant on native protein resources from both marine and terrestrial ecosystems than domestic animals and horticultural plants due to the time necessary to establish a extensive crops and animal populations. We also hypothesize that males would have been eating different foods compared to females, specifically these ‘high-status’ animal products because of the suspected ‘ranked’ nature of Lapita communities.

### Stable isotope analysis for paleodietary reconstruction

The stable isotope analysis of carbon (δ^13^C), nitrogen (δ^15^N) and, more recently, sulfur (δ^34^S) from human skeletal and dental remains is a well-established method of reconstructing ancient diets [52]–[55]. Stable isotopes are measured in ratios (^13^C /^12^C, ^15^N/^14^N, and ^34^S/^32^S) relative to a standard (PDB for carbon, AIR for nitrogen and VCDT for sulfur), expressed in parts per thousand or per mil (‰) and designated by the delta notation (δ). The rate of bone remodeling is slow for adults and therefore the stable isotope ratios of bone collagen are thought to represent at least the last 10–15 years of diet prior to death [56].

Carbon stable isotope ratios are used to address questions regarding marine and terrestrial food exploitation. Additionally, carbon stable isotope ratios are used to identify the consumption of certain types of plants that display different photosynthetic pathways (i.e. C_3_, C_4_ and CAM plants). This is possible because marine systems and C_4_ plants are enriched in the heavy stable isotope of carbon (^13^C) compared with terrestrial systems and C_3_ plants, respectively [54], [57].

There is a stepwise enrichment in ^15^N with every successive trophic level. Herbivores display higher δ^15^N values than the plants in their diet and carnivores display a subsequent enrichment in ^15^N in their bone collagen compared with the herbivores that they consume [58], [59]. Nitrogen stable isotope ratios are used in conjunction with carbon stable isotope ratios to differentiate between terrestrial and aquatic dietary resources. Marine, estuarine, and freshwater systems typically exhibit more steps in their respective food chains (i.e. more trophic levels) than terrestrial systems and correspondingly display higher δ^15^N values [54], [60]–[62]. Plants and organisms that utilize nitrogen-fixing bacteria such as legumes, seagrasses, mangroves, and cyanobacteria in coral reefs display low δ^15^N values [63]–[66].

Used in conjunction with carbon and nitrogen stable isotope ratios, the analysis of sulfur stable isotope ratios has become increasingly popular as a means to differentiate between aquatic and terrestrial dietary resources [27], [40], [67]–[71], and has also been used to identify migrants within a skeletal sample [72]–[74]. The δ^34^S value of human bone collagen is a reflection of local environmental sulfur isotope ratios [53], [75]. Sulfur enters terrestrial ecosystems from a number of sources including the local geology (e.g. pyrites), microbial processes in the soil and the atmosphere (wet and dry atmospheric deposition). Inorganic sulfates enter the local food web with little fractionation after they are converted into organic forms by plants [53], [73], [76]. Terrestrial systems may display a wide range of δ^34^S values (−5 to +10‰) [77], depending on the underlying geology, their proximity to the ocean (discussed below), and microbial processes in the soil [78], [79]. Freshwater systems display highly variable δ^34^S values (−22‰ to +20‰) [80], [81] as a result of the action of anaerobic bacteria in lake and river sediments [82]. Oceanic sulfate displays a consistent mean δ^34^S value of ∼20‰ [83] due to the continual mixing of oceans. Marine organisms can therefore be distinguished by their higher δ^34^S values in comparison to terrestrial organisms [80], [84]. However, the δ^34^S values of coastal terrestrial food webs may be elevated as a result of marine derived precipitation and sea-spray, known as the ‘sea-spray effect’ [33], [85], [86].

Human and faunal bone collagen stable isotope values from one site are directly comparable, taking into account trophic level differences for carbon, nitrogen and sulfur stable isotopes. It is widely accepted that trophic differences between consumer and prey nitrogen stable isotope ratios are between 3–4‰ [59], [87], although recently a larger offset of 6‰ has been suggested for humans in certain circumstances [88]. Trophic level enrichment of δ^13^C values has been found to be small, about 0–2‰, between consumer and prey collagen values [89]. The trophic enrichment of δ^34^S in bone collagen is not well established but is thought to be relatively small (<1.0‰) [53].

Nitrogen and sulfur stable isotope ratios are only representative of dietary protein as carbohydrates and lipids do not contain these elements [61]. Carbon from amino acids in dietary protein is preferentially routed to synthesize bone collagen, although some carbon from other macronutrients (e.g. lipids and carbohydrates) may also be utilized [90]–[93]. As a result, low protein foods (i.e. plant foods) may be underestimated in the diet when interpreting carbon stable isotope ratios [94]. The diet-tissue spacing of bone collagen δ^13^C values has been observed to be approximately +5‰ if the diet is monoisotopic (e.g. all components are from a C_3_ terrestrial ecosystem), but variations in diet-tissue spacing may occur as a result of the isotopic composition of the dietary macronutrients [90], [91], [95].

It is important to recognize that the interpretation of the proportions of certain foods in the diet from bone collagen isotope values is not straightforward. Establishing 100% terrestrial and 100% marine diets to use as “endpoints” is difficult because, as Müldner and Richards [96: 8] state, “uncertainty about the range of animal and plant foods consumed, their digestible protein content (which may vary according to their mode of preparation), as well as about the exact magnitude of the trophic level enrichment, means that where such end points are estimated, the likely errors involved must be considered”. The calculation of a 100% marine diet is difficult in the Pacific Islands because there are multiple sources of marine foods with a wide-range of δ^13^C values and C_4_ plants such as sugar cane may also have been eaten. For these reasons we refrain from estimating exact proportions of dietary protein in this paper.

### The site and the skeletal sample

**Figure 2:**
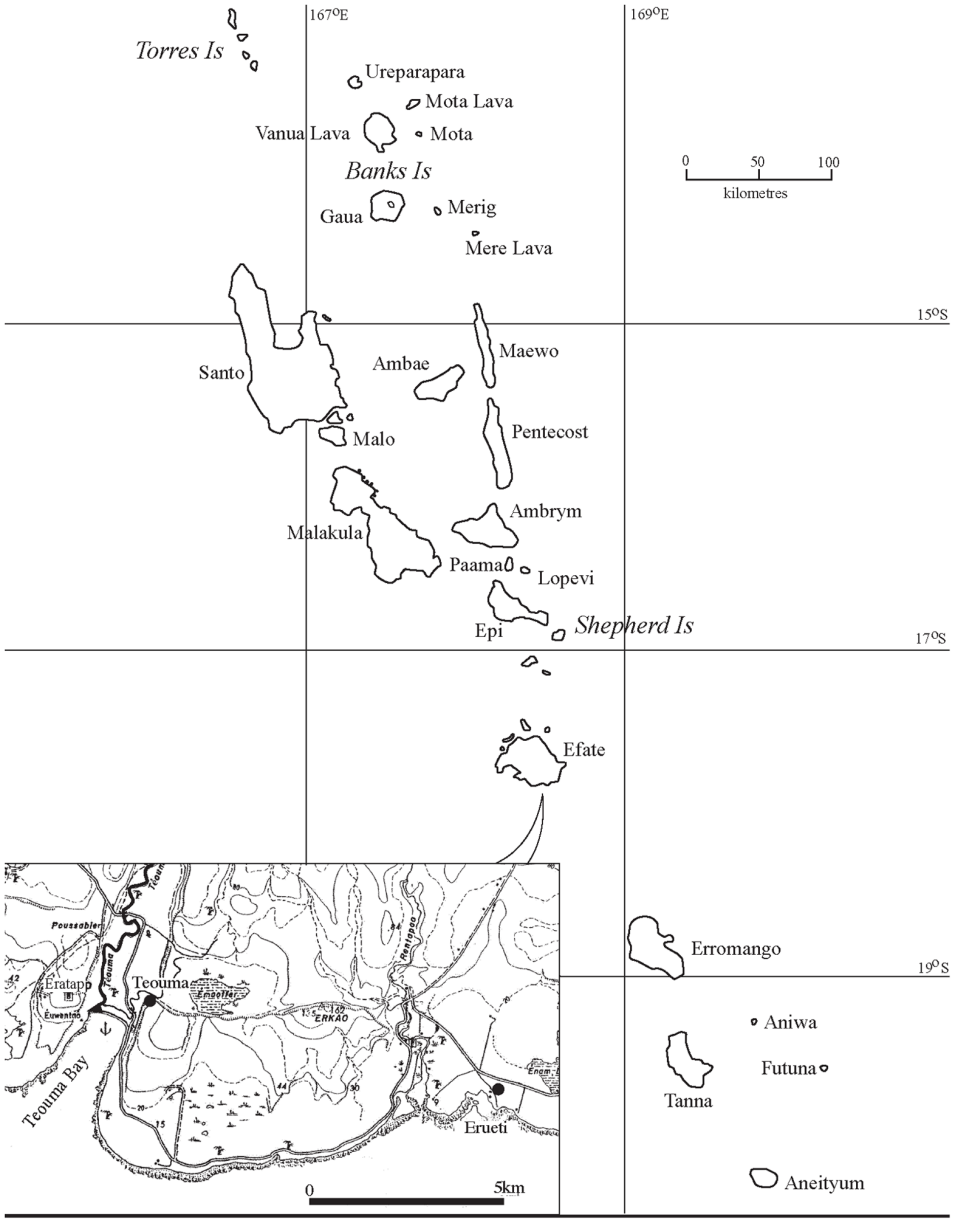
Map of the location of the Teouma site on Efate Island, Vanuatu.

The Teouma site is located close to modern day Port Vila on Efate Island, Vanuatu. Three thousand years ago the Teouma site would have been adjacent to the sea on the edge of a large bay, but as a result of tectonic uplift the site is now located approximately 800 m from the current foreshore [97] (fig. 2). The site was first excavated in 2004 as a result of the discovery of sherds of Lapita pottery during quarrying for the construction of a prawn farm [31], [97]. Sixty-eight graves, which contained single and multiple interments of around one hundred individuals, were recovered from the Lapita cemetery. All the individuals interred in the main cemetery date to the earliest phase of the Lapita settlement of Vanuatu (ca. 3000 BP) [31], [98]. A complex multi-stage burial ritual has been identified at the site, involving the manipulation of the bodies after death, the removal of bones and, in one case, cremation [99], [100].

The stratigraphy of the site is comprised of an ancient volcanic tephra covering the upraised karstic reef terrace and the former rolled-coral upper beach. Lapita burials are cut into the tephra layer, filling solution holes in the reef and upper beach. Up to 50 cm of midden associated with the Arapus and Early Erueti phases (ca. 2800–2500 BP) (Layer 2) overlies and post-dates the Lapita-associated burials (Layer 3). It is thought that this midden was deposited some time after the use of the site as a cemetery had ceased [31], [97]. Capping the archaeological deposits is a black tephra-rich soil from subsequent prehistoric volcanic eruptions. The unique depositional nature of the Teouma site, with the alkaline coral reef neutralizing the potentially acidic volcanic tephra, has led to the exceptional preservation of the skeletal and faunal material.

### Prehistoric diet at Teouma: the archaeological and ecological evidence

The Teouma site is located in an ecologically diverse area that is near to rainforest, lagoon, coral reef, freshwater, mangroves, and swamp ecosystems, all of which would have provided potential food resources for the site's inhabitants [31]. The analysis of the Teouma archaeofaunal assemblages is currently underway, but a number of terrestrial endemic and native species have been identified from archaeological contexts at the site and at other contemporary habitation sites in Vanuatu. Some of these species inhabit Vanuatu today (e.g. bats and a variety of smaller bird species), but some taxa were hunted to extinction during prehistory, such as a land-based crocodile (*Mekosuchus kalpokasi*), a number of flightless birds including a large megapode, several species of bat and a giant tortoise (? *Meiolania damelipi*) [101]–[103]. The extreme predation of fragile native and endemic species is a common feature of initial human occupation of the islands of Remote Oceania [101], [104]–[108]. Chickens, pigs and two rat species (*Rattus exulans* and *Rattus praetor*) comprised the domestic and commensal animals at Teouma (S. Hawkins unpublished data).

It remains uncertain exactly when many exotic domesticated plant species were introduced by humans to the islands of Remote Oceania, including Vanuatu. However, many cultivars were established in Vanuatu prior to European contact, and it has been speculated that a number of them were carried with the initial Lapita colonists [109]. In terms of the plant foods available, the tropical rainforest surrounding the Teouma site would have provided a suitable area for swidden gardening, a process of removing stands of rainforest for the fallow rotation of crops [109]. The underlying soil on South Efate is nutrient-rich because of the volcanic tephra deposited before the arrival of Lapita populations [31]. Although there is no direct evidence thus far regarding horticulture at Teouma (the analysis of microfossils from human dental calculus is currently underway), microfossil remains from sites in Northern Vanuatu do provide evidence of horticulture in Vanuatu in Lapita and post-Lapita settlements [20]–[22], [24]. Starch grains, phytoliths, calcium oxalate crystals and xylem cells have been used to identify imported cultivated species including *Musa* (banana), Araceae (aroids) and yam (*Dioscorea* spp.) in Lapita or immediately post-Lapita layers on the islands of Uripiv and Vao off the coast of northeast Malakula and on Epi Island. Some of these sites also show evidence of a decrease in Arecaceae (palms) and an increase in Poaceae (grasses), trends indicative of vegetation clearance likely associated with human settlement and horticultural activity [20]–[22].

Although there would have been some edible plants available when people arrived in Vanuatu, the distribution and abundance of these species is unknown [109], [110]. A majority of the wild foods probably available during the colonization of Remote Oceania would have consisted of leafy greens, fruits and nuts [111]. Throughout the Pacific Islands, these types of food are typically used to supplement meals, whereas starchy vegetables such as yam and taro (in addition to breadfruit, *Artocarpus altilis*) comprise the major constituents of meals [109], [112]. Native plants used to supplement meals in Vanuatu today include wild ferns, *Polyscias scutellaria*, nuts including *Canarium* spp., and fruits such as *Gnetum gnemon* and *Ficus* spp. [110], [113]. It must be noted that the large size of fruits and nuts and high yields of arboricultural crops found today throughout the Pacific are a result of thousands of years of selection and cultivation by humans [113]. Remains of coconut (*Cocos nucifera*) dating to ca. 5000 BP have been found on Aneityum Island in the south of Vanuatu [114] and coconut would likely have also been present on Efate when Lapita populations first arrived. Vanuatu was also the center of domestication of kava (*Piper methysticum*), but this plant is used for its medicinal and psychoactive properties and is not a food item [115]. Of the numerous starchy root vegetables eaten regularly in Vanuatu, the five-finger yam (*Dioscorea pentaphylla*) is the only edible tuber suggested to be endemic to the island group and is not a major dietary item anywhere today [109]. Certain non-native plants used for medicinal purposes or eaten infrequently today in Vanuatu (e.g. in times of famine or severe food shortage), such as cycas (*Cycas seemannii*), sago (*Metroxylon* sp.), kudzu (*Pueraria lobata*), wild yam (*Dioscorea nummularia*), and *Cordyline* spp. may have been consumed more regularly during prehistory [111], [116], [117] but were replaced when less labor intensive, higher yielding crops were established.

## Materials and Methods

### Ethics Statement

All necessary permits were obtained for the described study, which complied with all relevant regulations. Research agreements were signed between S. Bedford and M. Spriggs, the Vanuatu Kaljoral Senta and the Vanuatu Cultural Council, which included permission to excavate the Lapita site at Teouma, Efate Island, Vanuatu. The permits for exporting the human and faunal remains from the Teouma archaeological site were issued by the Vanuatu Kaljoral Senta and the Vanuatu Cultural Council (export permit numbers 11/VLI/04, 04/VLI/05, 14/VLI/06, 17/VLI/08, 08/VLI/09 and 09/VLI/2010). The prehistoric human remains are currently curated at the Vanuatu Kaljoral Senta, Port Vila, Vanuatu. The prehistoric faunal assemblage is curated by the School of Archaeology and Anthropology at the Australian National University, Canberra, Australia with the exception of the excess bone left over from stable isotope analysis (n = 73), which is stored in the Department of Anatomy, University of Otago, Dunedin, New Zealand. Modern plant and animal samples from Vanuatu were collected on private land with permission from private landowners and chiefs. Only modern wild animals (no domestic species) were sampled and no protected species were collected. All samples imported from Vanuatu (modern) or Australia (prehistoric) to the transitional facility at the University of Otago, Dunedin, New Zealand, were sent under the MAF (Biosecurity New Zealand Ministry of Agriculture and Forestry) Permit to Import Restricted Biological Products of Animal Origin (2011 permit number 2011041970) and the Permit to Import Laboratory Specimens (2011 permit number 2011041930). The modern plant and animal material is currently stored in a PC2 laboratory at the Department of Anatomy, University of Otago, Dunedin, New Zealand. All specimen numbers are detailed in full in Tables S1, S2 and S3.

### Materials and Methods

Cortical bone was sampled from fifty-one adult individuals from Teouma. The carbon and nitrogen stable isotope values from thirty-three of these individuals have been previously reported in Valentin et al. [32] (Table S1). The age and sex of the Teouma adults were estimated using standards found in Buikstra and Ubelaker [118].

Prehistoric faunal samples from pig (*Sus scrofa,* n = 18), chicken (*Gallus gallus*, n = 5), tortoise (? *Meiolania damelipi*, n = 16), sea turtle (n = 3), and fruit bat (Pteropodidae, n = 20) were analyzed to create the prehistoric dietary baseline for this study. In addition to the samples collected for this study, previously published stable isotope data from eleven prehistoric animals from Teouma (n = 2 pigs, n = 2 chickens, n = 2 tortoises, n = 2 rats, n = 2 fruit bats, and n = 1 reef fish) were also included in the dietary baseline to increase the sample size [32], [102], [119] (Table S2). Additionally, modern plants (n = 68) and animals (n = 30) were collected from Efate Island and northeast Malakula, Vanuatu (Table S3), and analyzed for carbon, nitrogen, and sulfur stable isotope ratios to provide further environmental baseline data. To account for the global decrease in ^13^C after the Industrial Revolution (i.e. the Suess effect), modern Pacific island plant and animal δ^13^C values within the baseline data were corrected by +1.5‰ for terrestrial and +.86‰ for marine systems [26], [120]. Very few aquatic animals from Vanuatu could be sampled for this study; therefore, published isotope values of Pacific island marine fish bones, seaweed (macroalgae), marine turtle bones and freshwater organisms were also included in the dietary baseline [27], [30], [94], [121]–[125].

Modern bone, plant, and shellfish flesh samples were rinsed in distilled water and defatted in methanol and chloroform (2∶1 v/v) until lipids were removed at the University of Otago (Dunedin, New Zealand) [126]. Plant and flesh samples were then lyophilized and weighed before isotopic analysis. The method used to extract the collagen from the bone samples prepared at the University of Otago followed the Longin [127] method modified by Brown et al. [128] and Collins and Galley [129] described in detail in Kinaston et al. [33]. Collagen was purified from the bone samples prepared at Aix-Marseille University (Marseille, France) using the Longin [127] method modified by and described in Bocherens et al. [130]. The purified “collagen” was analyzed by EA-IRMS at Iso-Trace (Dunedin, New Zealand) or Iso-Analytical (Cheshire, United Kingdom) (cf. Table S1). Analytical error was routinely ±0.10‰ for δ^13^C,±0.20‰ for δ^15^N, and ±0.30‰ for δ^34^S.

## Results and Discussion

The following sections present the stable isotope data, offering interpretations of the isotope values within the context of what is known about Vanuatu island ecology, in addition to addressing the hypotheses.

### Preservation of archaeological samples

An initial crucial step in the interpretation of these data was to assess the quality of the collagen to determine which samples could be included in subsequent analyses. In contrast to Valentin et al. [32] – who excluded ten individuals from their statistical analyses and dietary interpretations because the samples did not reach specific collagen quality indicators (i.e. wt %C higher than 30.0%, wt%N higher than 11.0%) [131] although their C:N ratios were between 2.9 and 3.6 described by DeNiro [132] as well-preserved – we have only rejected the two individuals (burials 63 and 64) with C:N values outside of 2.9 and 3.6. As outlined in White and Schwarcz [133] a significant correlation between C:N ratios and 1) δ^13^C values and 2) δ^15^N values may be indicative of post-depositional diagenetic alteration of collagen. No significant correlations between these variables were observed for the ten individuals removed by Valentin et al. [32] and burials 51 and 53 from the later analyses (n = 12; Spearman's *r* = 0.233, *p* = 0.469; Spearman's *r* = −0.201, *p* = 0.531, respectively) or the other ‘well-preserved’ individuals (n = 44; Spearman's *r* = −0.004, *p* = 0.978; Spearman's *r* = 0.269, *p* = 0.078). Thus, the adequate C:N ratios and the lack of a significant correlation between these variables in addition to the fact that any ‘outlier’ displayed wt %C over 30% and wt %N over 11% [131] suggest that the collagen of all the individuals except two (burials 63 and 64) was not affected by diagenetic alteration or post-depositional contamination. (Table S1). Ten of the human collagen samples analyzed for sulfur stable isotope ratios did not reach the sulfur collagen quality criteria outlined in Nehlich and Richards [134] (C:S ratio of 600±300, a N:S ratio of 200±100 and a wt %S lower than 60%) and were removed from subsequent analyses.

Of the most recently analyzed prehistoric faunal samples, all the animals reached the quality collagen indictors used in previous publications with the exception of two animals (a tortoise and a sea turtle), which displayed C:N ratios higher then 3.6 and were removed from the following analyses. The pig (*Sus scrofa*) and reef fish (Scaridae) that were removed from the Valentin et al. [32] study because they did not meet the collagen quality criteria were included in this study as their C:N ratios were between 2.9 and 3.6. All of the samples analyzed for sulfur stable isotope ratios reached the collagen quality indicators outlined in Nehlich and Richards [134] and described above. It must be noted that non-mammalian (e.g. tortoise) and mammalian bone collagen may display naturally differing collagen wt %C, wt %N and wt %S compositions. Therefore collagen quality indicators may be species-dependent [131], [134], [135].

### Dietary baseline: modern plants and animals

The stable isotope ratios of modern plants and animals found in the local food web were used to develop a dietary baseline to interpret the potential diet of the human and prehistoric fauna. The collection location, raw data and the summary statistics for the carbon, nitrogen and sulfur stable isotope ratios of the modern floral and faunal samples analyzed in this study are located in Supplementary Table S3 and Table 1 respectively.

**Table 1:** Descriptive statistics (mean ±1 SD) for δ^13^C, δ^15^N and δ^34^S values of modern plant and animals collected from Vanuatu.

Sample	n	δ^13^C (‰)	±1 SD	δ^15^N (‰)	±1 SD	n	δ^34^S (‰)	±1 SD
All C_3_ plants	62	−26.2	2.1	3.9	2.0	8	9.5	4.0
C_3_ plants (no nuts)	49	−26.3	2.2	3.6	1.7	5	8.0	3.7
C_3_ nuts	13	−25.6	2.0	5.1	2.4	3	11.9	3.8
Fruit bat bone	1	−21.7		7.1		1	11.0	
C_4_ plants	3	−12.5	0.1	3.9	1.0			
Seagrass	3	−9.3	4.8	1.2	0.5	1	14.0	
Mangrove shellfish	3	−26.1	2.5	−2.0	6.5	2	−8.7	18.0
Mangrove crabs	2	−24.1	0.0	2.5	0.6	1	9.4	
Marine shellfish	15	−10.1	4.7	5.5	2.0	1	14.9	
Sardine bone	2	−14.3	0.0	5.8	0	1	17.2	
Sphyraenidae bone	2	−12.3	0.1	6.0	0.3			
Parrotfish bone	2	−9.7	3.8	5.4	3.1	1	17.6	
Tuna bone	2	−13.0	0.1	7.3	0.3	1	18.2	
Marine turtle bone	1	−9.7		9.2		1	13.6	

**Figure 3:**
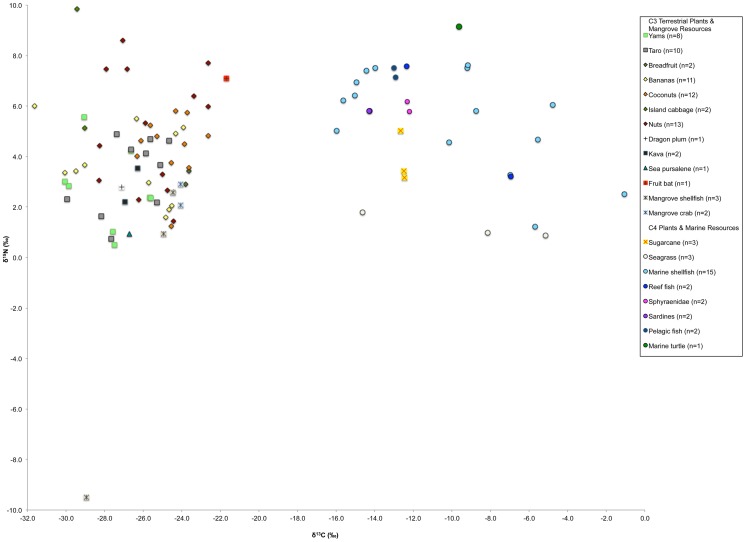
Modern plant and animal δ^13^C and δ^15^N values from Vanuatu.

Figure 3 details the carbon and nitrogen stable isotope values of the modern plants and animals from Vanuatu that were analyzed for this study. The modern δ^13^C and δ^15^N values of C_3_ and C_4_ plants from Vanuatu are similar to those found in the Marianas [94] and the Cook Islands [121]. Using a two-sample t-test assuming unequal variance (Levene's test *p* = 0.037), there was a tendency for δ^15^N values of the nuts to be higher than the non-nut C_3_ plant species (diff 1.5‰, *p* = 0.053). The δ^13^C and δ^15^N values of the modern fruit bat confirm that this animal ate fruits, flowers, nectar and insects from a C_3_-based terrestrial environment.

Seagrass δ^13^C and δ^15^N values were similar to those observed by Fry et al. [136] and Keegan and DeNiro [63]. Some C_3_ marine plants, including seagrasses and coral reef algae, have demonstrated δ^13^C values similar to plants with a C_4_ photosynthetic pathway and this is the case for the *Thalassia hemprichii* and *Syringodium isoetifolium* species analyzed in this study [64], [137], [138]. Coral reef, seagrass and mangrove ecosystems display low δ^15^N values as a result of a prevalence of N**_2_**-fixing bacteria in these environments [60], [63], [64]. Accordingly, the shellfish (n = 3) and crabs endemic to mangroves (n = 2) displayed low average δ^15^N values ranging from −9.5‰ to 2.9‰ but, as a result of their C_3_ mangrove environment, exhibited terrestrial δ^13^C values ranging from −28.9‰ to −24.1‰.

The δ^13^C values of the marine shellfish from environments outside the mangroves (n = 15) were, on average, lower than the δ^13^C values of limited number (n = 7) of tropical species analyzed in other studies, although their δ^15^N values were similar [26], [125]. The variation between studies is likely a result of the small number of samples analyzed, species-specific isotope variation, environmental factors and differences in defatting protocols. Eight marine fish (two reef fish, two fish with variable feeding areas, two pelagic tuna and two pelagic sardines) were sampled. On average, the reef fish and low trophic level pelagic species (sardines) displayed the lowest δ^15^N values; the higher trophic level deep-water species (tuna) displayed the highest δ^15^N values; and the species that utilized both environments displayed intermediate values. It must be noted that the pelagic fish in this study (all tuna) displayed lower δ^15^N values compared with pelagic fish and sea mammals analyzed in other studies [27], [30], [94], [123], likely a reflection of the specific taxa, fish size and feeding environment. Otherwise, the carbon and nitrogen stable isotope values of the marine fish species analyzed in this study generally agreed with those observed by other Pacific island stable isotope studies that analyzed tropical fish bone [26], [27], [30], [121]–[123]. It is difficult to compare the isotope values of the modern turtle bone with those presented by other studies because of the potential isotopic variation resulting from age-specific foraging strategies and migratory patterns of species such as *Chelonia mydas* (green turtle) and *Eretmochelys imbricate* (hawksbill turtle) [139], [140]. The high δ^13^C values of the modern marine turtle bone (−9.7‰) in this study indicate that this animal likely fed in ^13^C enriched environments, such as seagrass meadows and coral reefs.

The average δ^34^S value of the modern terrestrial plants ranged from 3.7‰ to 15.7‰ (average 9.5‰±4.0‰). As previously mentioned, marine-derived precipitation and sea-spray can raise the δ^34^S values of terrestrial plants and animals, an effect of marine sulfates (averaging ∼21.0‰) entering coastal food webs [53], [83]. The elevated δ^34^S values of the modern Vanuatu plants are likely a result of this sea-spray effect. The large variation in terrestrial δ^34^S values is most likely associated with garden location, with the plants displaying the low δ^34^S values originating from inland gardens further away from the coast. The δ^34^S value of the modern fruit bat (11.0‰) further indicates that marine-derived sulfates have affected the local terrestrial food web as these animals consume purely terrestrial resources. Elevated terrestrial plant and animal δ^34^S values have been observed in other Pacific island paleodietary studies [26], [27], indicating that sea-spray may influence terrestrial sulfur stable isotope ratios and thus potentially affect subsequent dietary interpretations.

Primary producers at the base of marine food webs exhibit δ^34^S values ranging from +17‰ to +21‰ [80], [84]. All of the modern fish analyzed for sulfur stable isotope ratios fell within this range. The lower δ^34^S values of the seagrass (14.0‰), marine turtle (13.6‰), marine shellfish (14.9‰), crab (9.4‰) and mangrove shellfish (average −8.7‰) analyzed in the current study are likely a result of environmental conditions such as the proximity to mangroves and the effect of terrestrial detritus [141], [142]. Plants living in anaerobic sediments found in mangroves and seagrass meadows utilize ^34^S-depleted sulfides and display low δ^34^S values [143] and the action of sulfate-reducing bacteria in anoxic marine sediments has been suggested as a reason for low δ^34^S values of marine mammals in northern Europe [141]. The exceptionally low δ^34^S value of one species of mangrove bivalve (*Anodontia philippiana*) (−21.5‰) is likely a reflection of its chemosymbiotic relationship with sulfide-oxidizing bacteria living in its gills [144] and, as these species are widespread [145], should be taken into consideration when assessing diet using sulfur stable isotope ratios where relevant. We do not believe that modern pollutants have influenced the marine or terrestrial δ^34^S values [86] as the areas of Vanuatu where these samples were collected are not industrialized.

### Dietary baseline: Prehistoric fauna

The stable isotope values of the prehistoric fauna analyzed from the site of Teouma provide further baseline information about the foods available to the humans living in the settlement. An assessment of the diet of domesticated animals also has the potential to provide information about the management of these species, especially pigs, and about how the humans and animals interacted within their shared environment. A potential outcome of analyzing the wild faunal resources is that their isotope values are a reflection of the ecological niches in which they live that may vary over time partly as a consequence of human impact on the environment.

**Figure 4:**
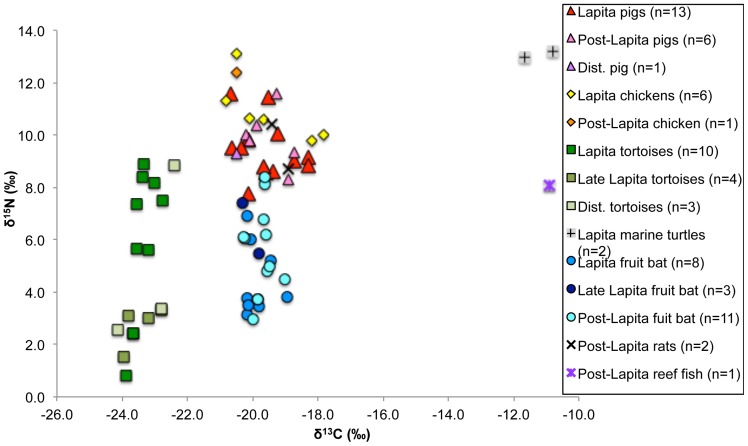
Prehistoric faunal δ^13^C and δ^15^N values from Teouma.

The isotope results for the fauna from Teouma are located in Supplementary Table S2 and Figure 4. Summary statistics for the prehistoric animals from Teouma are presented in Table 2. The mean (±1 SD) δ^13^C value of the prehistoric fruit bats (−19.8‰±0.4‰, n = 22) confirmed that these animals were primarily eating foods from C_3_-based environments [146]. The prehistoric fruit bat values are in agreement with other studies that suggest the δ^13^C value of a purely terrestrial diet to be ∼−20.0‰±1.0‰ [35], [123]. There was a large variation in fruit bat δ^15^N values, ranging from 3.0‰–8.4‰ which may be the result of specific feeding strategies as some environments, such a mangroves, can exhibit variable δ^13^C and δ^15^N values depending on their location relative to the coast [147]. However, we suggest that a likely cause of the observed variation in δ^15^N values is the result of the inclusion of insect-eating bats in our study. Megabats (Pteropodidae) commonly feed on fruit, nectar and flowers but are also known to be insectivorous [146], [148]. There are twelve species of bat found throughout Vanuatu today, three of which are megabats [149], [150]. However, several distinct and as yet unidentified fruit bat species from the Pteropodidae family have been identified from the Teouma, Uripiv and Vao (northeast Malakula, Vanuatu) archaeological sites. Now-extinct species are most certainly present in these assemblages and variations in bat δ^15^N values could reflect differences in ecological adaptations from these various bat species (S. Hawkins, unpublished data).

**Table 2:** Descriptive statistics (mean ±1 SD) for δ^13^C, δ^15^N and δ^34^S values of the prehistoric fauna from Teouma.

Animal	Layer	n	δ^13^C (‰)	±1 SD	δ^15^N (‰)	±1 SD	n	δ^34^S (‰)	±1 SD
Pig	All Layers	20	−19.6	0.7	9.6	1.1	18	10.9	0.8
Pig	Post-Lapita	6	−19.5	0.6	9.9	1.1	4	10.9	0.9
Pig	Lapita	13	−19.6	0.8	9.4	1.1	13	11.0	0.9
Pig	Disturbed	1	−20.5		9.3				
Chicken	All Layers	7	−19.7	1.1	11.1	1.2	5	12.2	0.8
Chicken	Post-Lapita	1	−20.5		12.4				
Chicken	Lapita	6	−19.5	1.2	10.9	1.2	3	12.2	0.8
Tortoise	All Layers	17	−23.4	0.5	4.9	2.8	4	11.0	0.8
Tortoise	Late Lapita	4	−23.4	0.5	2.7	0.8	1	10.4	
Tortoise	Lapita	10	−23.4	0.3	5.7	2.9	1	10.4	
Tortoise	Disturbed	3	−23.1	0.9	4.9	3.4	2	11.7	0.5
Marine turtle	Lapita	2	−11.2	0.6	13.1	0.1			
Rat	Post-Lapita	2	−19.2	0.4	9.6	1.2			
Reef fish	Post-Lapita	1	−10.9		8.1				
Fruit bat	All Layers	22	−19.8	0.4	5.2	1.6			
Fruit bat	Post-Lapita	11	−19.7	0.4	5.7	1.7			
Fruit bat	Late Lapita	3	−20.0	0.3	5.5	1.9			
Fruit bat	Lapita	8	−19.9	0.4	4.5	1.4			

The average δ^15^N values of the fruit bats increased by 1.0‰ from the Lapita to late Lapita period, but only 0.2‰ between the late Lapita and the post-Lapita periods (note that the sample sizes were too small to conduct statistical analyses). These differences may be a result of the human establishment of fruit-bearing arboricultural species with different δ^15^N values compared to native trees, vegetation change resulting from climate change or human disturbance [151], or it may just be that more insectivorous bats were present in the later assemblages. The presence of nursing or juvenile individuals in the sample, which would result in the enrichment of ^15^N in the infant bats' tissues, could also be responsible for the observed variation of δ^15^N values in these animals [148], [152].

The average δ^13^C values of the tortoise, another purely terrestrial species, are 3.6‰ lower compared with the average fruit bat δ^13^C values, which may be related to the ecological zones they utilized or possibly a physiological effect. Plants that grow under the forest canopy display low δ^13^C values as a result of recycling ^13^C-depleted CO_2_ from decomposing forest floor litter during photosynthesis, a phenomenon known as the ‘canopy effect’ [153], [154]. Thus, the consumption of plants under the rainforest canopy by tortoises may account for their low δ^13^C values. Nine of the tortoises displayed δ^15^N values lower than 4.0‰ (n = 3/10 from the Lapita assemblage, n = 4/4 from the late Lapita assemblage and n = 2/3 from the disturbed assemblage), whereas the other eight tortoises displayed values higher than 5.5‰ (n = 7/10 from the Lapita assemblage and n = 1/3 from disturbed layers). Although the sample sizes are small, this trend may be indicative of changing environmental conditions as a result of human settlement. The δ^13^C values of all tortoises were very similar (−23.4‰±0.5‰, n = 17) and therefore the variation in δ^15^N values is likely a result of the regular consumption of foods from different trophic levels. Higher trophic level foods could have included insects and freshwater organisms such as crustaceans, mollusks and freshwater fish. The tortoises that displayed the lowest δ^15^N values may have utilized the mangrove environment for food as their values were similar to mangrove shellfish and crabs. It is difficult to extrapolate on the dietary breadth of these extinct animals, but the variation in δ^15^N values may point towards age-related dietary variations or diverse foraging environments. Evidence from the ongoing faunal studies suggests that the Teouma human population was forced to target ever more-distant tortoise populations as high levels of predation led to depletion of resources closer to the site (S. Hawkins, unpublished data).

The domestic and commensal species displayed δ^13^C values that indicate that their diets were highly terrestrial (pig  = −19.6‰±0.7‰, n = 20, chicken  = −19.7‰±1.1‰, n = 7 and rat  = −19.2‰±0.4‰, n = 2), although some animals displayed a minor marine signal. These values suggest that they were either fed C_3_ terrestrial foods by humans, such as cultivated plants, or allowed to forage for food in the C_3_ environments surrounding Teouma, such as the rainforest. The limited marine input into both the Lapita and immediately post-Lapita domestic animal diets at Teouma may be indicative of some type of controlled feeding (even of wild resources) or penning away from the settlement, meaning they were unable to access marine food scraps. Another interpretation is that the marine foods consumed by humans such as shellfish and soft-shelled crustaceans left few edible remains.

The δ^15^N values of the domestic and commensal species are higher compared with the values of tortoises and fruit bats, suggesting consumption of these local animals (e.g. from human food scraps) or other terrestrial organisms such as insects [155], native animals such as small lizards and snakes [149] or human feces. Pigs practice caecotrophy and the regular consumption of human feces could result in the elevation of pig δ^15^N values [156]. The pigs from the immediately post-Lapita assemblage displayed slightly higher δ^13^C values (0.1‰) and higher δ^15^N values (0.5‰) compared with the same taxa in the Lapita assemblage. This dietary variation suggests that the pig population consumed resources from higher trophic levels in the later period. The post-Lapita chicken displayed a δ^15^N value that was 1.5‰ higher and a δ^13^C value that was 1.0‰ higher than the chickens (n = 6) from the Lapita period, although it is noted that these sample sizes are very small, preventing us from making meaningful inferences regarding temporal changes in chicken diet.

The prehistoric fauna from Teouma displayed a trend in δ^34^S value similar to the modern terrestrial plants and fruit bats. The land-based tortoise, a species undoubtedly consuming a terrestrial-based diet, exhibited δ^34^S values indicative of the sea-spray effect (11.0‰±0.8‰, n = 4). The δ^34^S values of the domestic species (pigs and chickens) all ranged between 9.7‰ and 13.0‰. The δ^13^C values of the domestic animals are representative of a predominately terrestrial-based diet, further supporting the suggestion that the sea-spray effect influenced their δ^34^S values. As the site was directly on the coast, this result is not entirely surprising, but does indicate that all species were affected similarly.

The δ^15^N values of the domestic animals are comparable with the highest values for the bats or are elevated compared to the bats indicating they were eating terrestrial resources from higher trophic levels. The domestic animal isotope data may be indicative of controlled feeding of C_3_ horticultural foods and terrestrial animal remains as hypothesized by Valentin et al. [32] but could also suggest that pigs and chickens relied heavily on foraging for wild foods, especially insects, and human feces. If the latter is the case, the data support the proposition by Clark et al. [157: 9] that during colonization the low population density and limited established garden areas would allow for “the presence of a free range scavenging pig population under benign environmental conditions”.

The presence of pigs at Lapita sites has previously been used as a proxy for horticulture [158], [159], but depending on the size of the pig and human populations horticulture is not necessarily a prerequisite for keeping pigs due to their ability to forage [160], [161]. Feral pig populations survive throughout Vanuatu, including on Efate Island, indicating that if released, pigs could have survived without the assistance of humans [149]. In New Guinea, pig husbandry methods range from the complete control of pig fodder by humans (sometimes dedicating a large proportion of vegetable crops to pigs) to completely relying on pigs to forage for their own food [161].

### Human diet at Teouma

**Figure 5:**
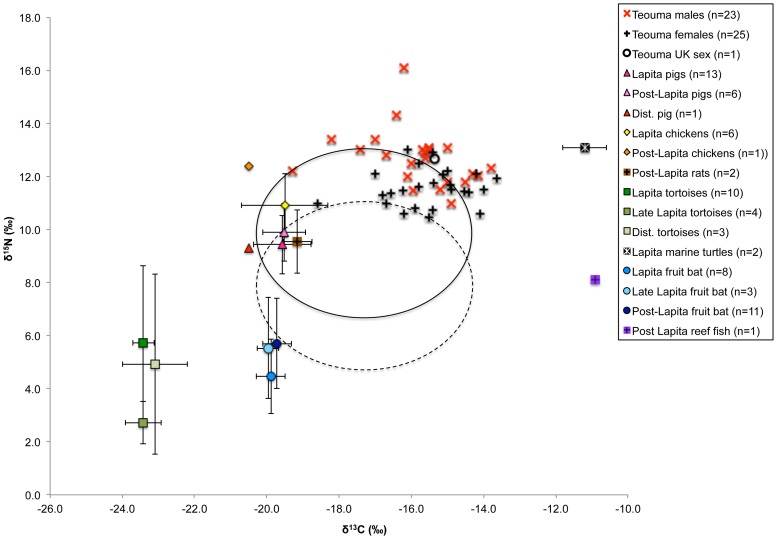
Human δ^13^C and δ^15^N values in relation to the prehistoric faunal remains from Teouma. The full and dotted circles designate a trophic effect of 1.0‰ for δ^13^C and 3.0‰ for δ^15^N and 1.0‰ for δ^13^C and 5.0‰ for δ^15^N respectively.

**Figure 6:**
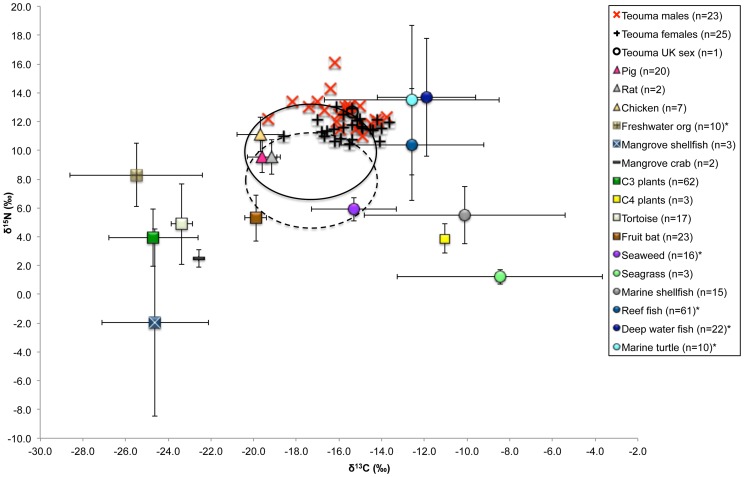
Teouma human δ^13^C and δ^15^N values in relation to a tropical Pacific island dietary baseline. The full and dotted circles designate a trophic effect of 1.0‰ for δ^13^C and 3.0‰ for δ^15^N and 1.0‰ for δ^13^C and 5.0‰ for δ^15^N respectively. Note that the modern δ^13^C values have been corrected for the Suess effect. The asterisks (*) denote the inclusion of faunal stable isotope data from other tropical Pacific paleodietary studies (see text for references).

The isotope data and demographic information for the humans are located in Supplementary Table S1. Summary statistics for the human data are located in Table 3. Figures 5 and 6 detail the human isotope values (δ^13^C and δ^15^N) in relation to the prehistoric faunal remains from Teouma and a wider Pacific dietary baseline, including the modern Vanuatu data.

**Table 3:** Descriptive statistics (mean ±1 SD) for δ^13^C, δ^15^N and δ^34^S values of the humans from Teouma.

Group	n	δ^13^C (‰)	±1 SD	δ^15^N (‰)	±1 SD	n	δ^34^S (‰)	±1 SD
All Adults	49	−15.7	1.2	12.1	1.0	14	11.3	1.3
Males	23	−15.8	1.3	12.7	1.1	10	11.5	1.3
Females	25	−15.5	1.1	11.6	0.7	4	10.9	1.1
Unsexed	1	−15.4		12.7				

It is generally accepted there is a trophic increase between 0–2.0‰ for δ^13^C and 3.0–6.0‰ for δ^15^N values between predator/prey bone collagen [88], [89]. The average human δ^13^C values were 3.9‰ higher compared with the δ^13^C values of the combined commensal species (pigs, rats and chickens) from all layers, indicating that they were eating these domestic species. A similar difference in δ^13^C values was observed between the humans and the endemic fruit bats (humans 4.1‰ higher), but this difference was much larger between the human and the tortoises (humans 7.7‰ higher). The average human δ^15^N values were 2.2‰ higher compared with the commensal species and much higher compared with the native species (humans 6.9‰ higher for the fruit bats and 7.2‰ higher for the tortoises). The low δ^13^C and δ^15^N values of the tortoise compared with the stable isotope values of the humans indicate these animals did not constitute a major dietary resource at Teouma during the cemetery phase.

It has previously been posited that land-based tortoises were hunted in preference to the marine turtles until their disappearance some 300 or so years after initial settlement as a result of human predation [102]. In fact, further faunal analysis has now shown that fruit bats, marine turtles, and tortoises were initially heavily targeted in close proximity to the site resulting in resource depletion and the subsequent subsistence change towards more mobile foraging tortoises (S. Hawkins, unpublished data). The δ^13^C and δ^15^N values of the humans support the faunal analysis evidence, specifically that the commensal animals and fruit bats constituted the major terrestrial protein resources at Teouma in the early cemetery phase.

The higher δ^13^C values of the humans compared to the commensal and native species indicates that ^13^C-enriched protein resources were also being consumed, most likely from the marine environment. Comparison with the dietary baseline data suggests that the people at Teouma were likely accessing resources from the reef in addition to sea turtle and possibly deeper-water species of fish. Inshore organisms are also likely to have been eaten, but their contribution is most visible when a larger trophic offset for δ^15^N (5.0‰) is used to interpret the human diet. The consumption of inshore organisms is typical of early Lapita sites, indicating foraging for nearby, easily accessible resources [7], [8], [162], [163]. In addition, reef and inshore species of fish are well-represented in Lapita middens across the western Pacific [30], [164]. Few remains of pelagic species or of deep-sea trolling fishhooks are found at Lapita sites (including Teouma), supporting the suggestion that the elevated δ^13^C and δ^15^N values are more likely a result of the consumption of marine turtle than deep-water fish.

The identification of sea turtle exploitation is difficult because of the large variation in stable isotope values for these animals [discussed in 32]. However, the consumption of marine turtle likely contributed to the elevated δ^13^C and δ^15^N values of the humans as these animals are well-represented in some early Vanuatu faunal assemblages [162], [165], including Teouma (S. Hawkins unpublished data).

Mangrove, freshwater and C_4_ plant resources (i.e. sugarcane) cannot be ruled out of the dietary interpretations but do not appear to contribute substantially to the diet at Teouma. Freshwater species display similar δ^13^C values to terrestrial ecosystems but higher δ^15^N values [125]. The freshwater stream running adjacent to the site may have provided access to freshwater fish, shellfish and other aquatic organisms [31], although, as a result of geographic isolation, both freshwater and mangrove species diversity is limited in Vanuatu [166], [167]. To address this issue it would be necessary to analyze modern freshwater organisms in the Teouma area.

Sugarcane (*Saccharum officinarum*), the only C_4_ terrestrial domesticated plant known in the Pacific Islands, or *Portulaca lutea,* an edible indigenous CAM/C_4_ plant [121], [168], may have also influenced the δ^13^C values observed in the Teouma sample. However, because sugarcane has a high carbohydrate and low protein content its consumption may not be well reflected in bone collagen stable isotope values [169].

**Figure 7:**
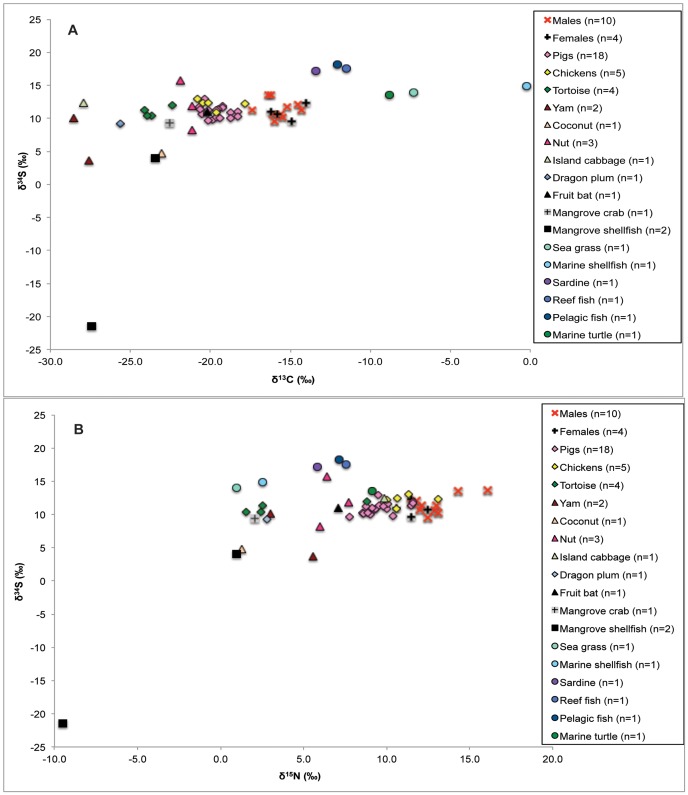
Teouma human, faunal, and floral δ^13^C and δ^34^S (A) and δ^15^N and δ^34^S (B) values. Note that the modern δ^13^C values have been corrected for the Suess effect.

The human sulfur isotope data are presented in Figure 7. The human δ^34^S values were 0.1‰ higher compared with the commensal species and 0.3‰ higher compared with the tortoise values. The δ^34^S values of the humans and fauna from Teouma are not consistent with the dietary patterns observed in the carbon and nitrogen stable isotope results. Confirming previous suggestions [26], [27], the δ^34^S values of the terrestrial plants and animals (both modern and prehistoric) in addition to the similarity of human and faunal isotopic values strongly indicate that the local food web was influenced by the sea-spray effect. As a result of this effect and large variations in the local ecosystem's δ^34^S values (e.g. mangrove organisms), it is difficult to use sulfur stable isotope ratios to differentiate between marine and terrestrial dietary components for the Teouma individuals and more generally for skeletal populations residing in coastal areas.

**Figure 8:**
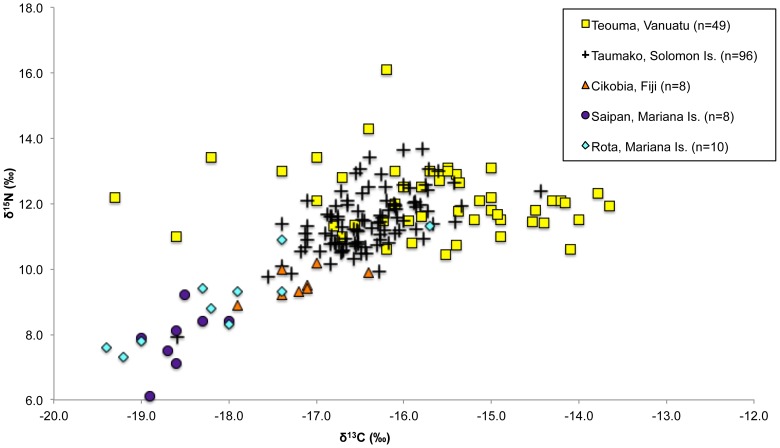
Comparison of Teouma δ^13^C and δ^15^N with other Pacific paleodietary studies (see text for references). Spearman's correlation of δ^13^C and δ^15^N values of adult humans from Teouma, Vanuatu (n = 49, *r*  = −0.210, *p* = 0.148), Taumako, Solomon Islands (n = 96, *r* = 0.541, *p*<0.001), Cikobia, Fiji Islands (n = 8, *r* = 0.651, *p* = 0.081), Rota, Mariana Islands (n = 10, *r* = 0.838, *p* = 0.002), and Saipan, Mariana Islands (n = 8, *r* = 0.723, *p* = 0.043).

The range in human δ^13^C and δ^15^N values in addition to the lack of any significant positive correlation for the sample (n = 49, Spearman's *r* = −0.210, *p* = 0.148) suggest that the population was subsisting on marine and terrestrial protein resources from similar trophic levels as one another, a different dietary pattern from that observed in later prehistoric Pacific island populations. For example, a statistically significant positive correlation between the δ^13^C and δ^15^N values of humans from the Polynesian outlier site of Taumako in the Solomon Islands (n = 96, Spearman's *r* = 0.541, *p*<0.001) and the range of δ^13^C and δ^15^N values (−16.4‰±0.6‰ and 11.5‰±0.9‰) indicated that their diet consisted of low trophic level plants (likely starchy root vegetables) and higher trophic level marine resources [33]. Similar dietary interpretations were posited for eight prehistoric/historic adult individuals from Cikobia in the Fiji Group, who exhibited low δ^13^C and δ^15^N values (−17.2‰±0.4‰ and 9.6‰±0.4‰ respectively), but maintained a positive, albeit insignificant, correlation between δ^13^C and δ^15^N values (n = 8, Spearman's *r* = 0.651, *p* = 0.081) [35]. In the Mariana Islands, the strong positive correlation between the bone collagen δ^13^C and δ^15^N values of the inhabitants of Rota (−18.1‰±1.1‰ and 9.0‰±1.3‰, n = 10, Spearman's *r* = 0.838, *p* = 0.002) and Siapan (−18.6‰±0.3‰ and 7.8‰±0.9‰, n = 8, Spearman's *r* = 0.723, *p* = 0.043) indicated that their protein diet consisted of a high proportion of starchy root vegetables and a lesser amount of marine protein [94] (fig. 8). Based on the analysis of the bone carbonate of the Marianas individuals it was suggested that C_4_ plants such as sugar cane or seaweeds were also consumed but these plant foods could not be identified using the stable isotope analysis of bone collagen because of their low protein content [94], [170].

The results from the human isotopic analyses support the interpretation of the dietary pattern proposed by Valentin et al. [32], specifically that the Lapita people from Teouma were primarily subsisting on reef foods, some higher trophic level marine animals (likely marine turtle), some inshore organisms and terrestrial animal protein resources, most importantly commensals and fruit bats. Comparison of the human isotope values with those of the fruit bat δ^13^C values (representing a purely C_3_ plant and insect diet) indicates that C_3_ plant foods did not constitute a major protein resource for the humans at Teouma. The lack of significant amounts of C_3_ plants in the human diet supports the assumption that horticulture was likely being established at this early stage and was less important to the overall diet compared with populations later in prehistory. The first hypothesis – that the people from Teouma were eating more local wild resources compared to domestic species and horticultural plants – is not falsified. However, in addition to these wild resources, domestic animals are also likely to have been important protein resources. The stable isotope data suggest that the subsistence strategy at the site was focused on foraging for wild marine and terrestrial resources while raising and eating domestic animals. These domestic species may have been raised using a free-range system of animal husbandry, reducing the competition between pigs and humans for the limited horticultural plant foods likely being grown at the time.

### Sexual differences in diet

Summary statistics for the males and females are presented in Table 3.

**Figure 9:**
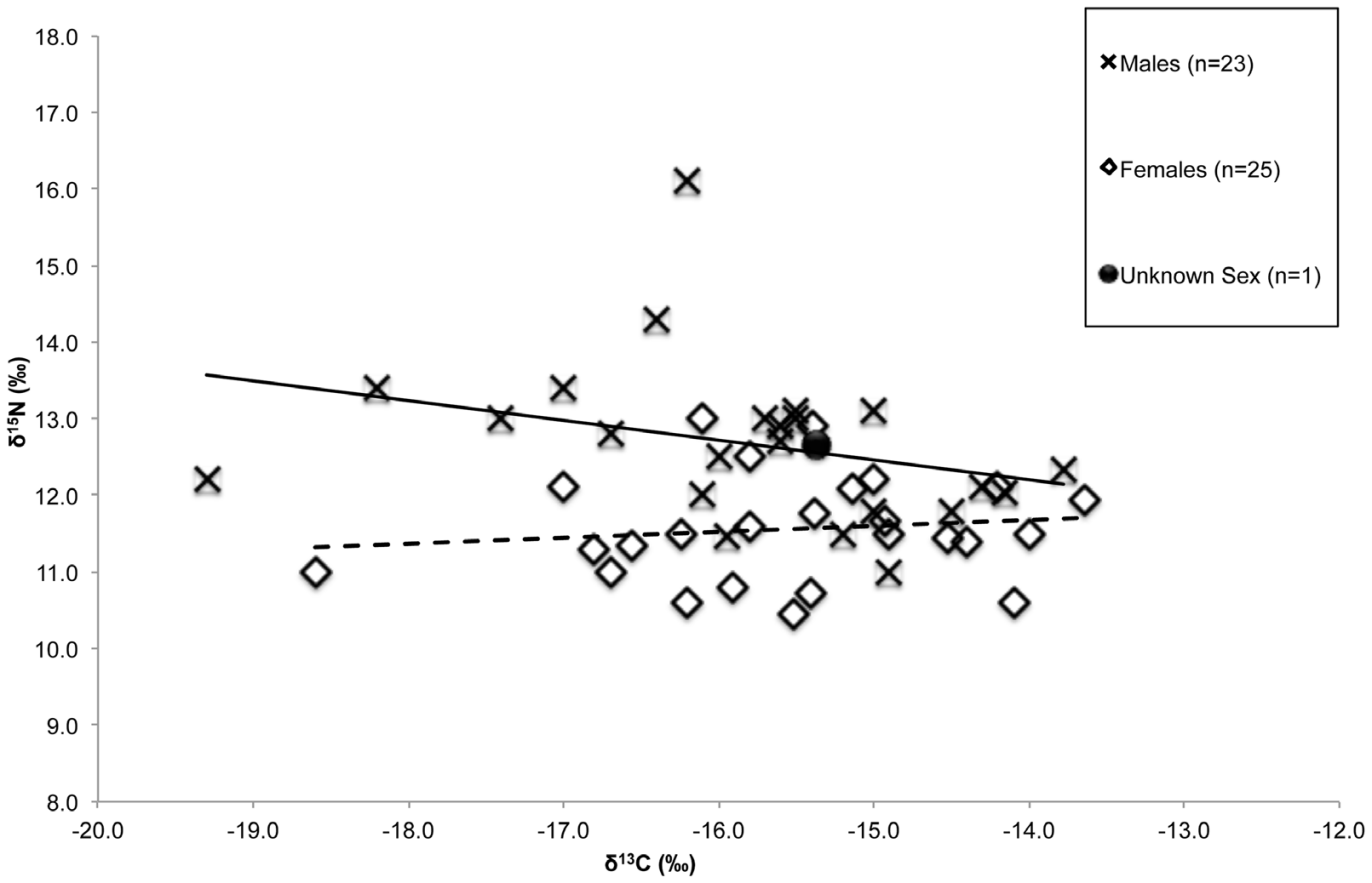
Teouma male and female δ^13^C and δ^15^N values. Spearman's correlation of δ^13^C and δ^15^N values of adult males (black line, n = 23, *r* = −0.500, *p* = 0.015) and females (dotted line, n = 25, *r* = 0.161, *p* = 0.443) from Teouma.

Compared with the females, the males displayed a larger range of both δ^13^C and δ^15^N values (female δ^13^C range −18.6‰ to −13.6‰ and male δ^13^C range −19.3‰ to −13.8‰, female δ^15^N range 10.4‰ to 13.0‰ and male δ^15^N range 11.0‰ to 16.1‰) (fig. 9). Using a two-sample t-test assuming equal variance (Levene's test *p* = 0.206), males displayed significantly higher δ^15^N values compared to females (12.7‰ *vs*. 11.6‰, *p*<0.001, male n = 23, female n = 25), but no significant differences were observed between the sexes in δ^13^C or δ^34^S values. Similar results were observed in Kinaston et al. [171], but the analysis of a larger sample of individuals from the site confirms that this dietary difference between the sexes is very important for understanding possible socio-cultural practices at this Lapita site.

Therefore, the stable isotope evidence supports the second hypothesis, that males and females had different diets. The stable isotope analysis indicates that males were consuming a more variable diet and higher trophic level protein resources than women. This trend suggests sexual differences in food consumption patterns. These dietary patterns may be representative of the differential roles of food acquisition between the sexes, with the males hunting and fishing while the women primarily gathered foods and foraged in the inshore environment. These gendered patterns of food acquisition have been observed in modern-day Pacific island communities [44], [112], [163], [172]–[174] and in late prehistoric populations from Papua New Guinea through to Polynesia [33], [40].

Alternatively, the observable difference between male and female diets may indicate that food distribution was culturally moderated and may suggest preferential treatment of males in the society, at least with regard to food access. In modern-day Pacific island cultures protein-rich animal foods are highly valued. Choice cuts of pig and chicken and relatively scarce marine foods (e.g. turtle and shark) are traditionally restricted to high status men within many communities [43], [47], [112], [163], [172]. It is possible that males at Teouma were considered of a higher status and thus receiving differential treatment to the women, expressed here in the unequal distribution of food resources, perhaps supporting the premise that Lapita societies were ranked in some way.

### Conclusions

The stable isotope analysis of the Teouma cemetery sample presented here constitutes the largest paleodietary study of Lapita period humans to date. In conjunction with the stable isotope values of the modern plants and animals and prehistoric fauna, these data collectively represent the most comprehensive assemblage of information on the diet and subsistence strategies of these enigmatic colonizers.

The interpretation of the paleodiet at Teouma in the context of a comprehensive dietary baseline has assisted in clarifying food procurement strategies during the initial Lapita colonization of Efate. The dietary pattern observed at Teouma supports a mixed subsistence strategy which included broad-spectrum marine resource foraging, the consumption of wild animals, especially fruit bats, and animal husbandry. These data indicate that the protein resources eaten by these early colonists were procured by a ‘strandlooping’ mode of subsistence, but also that domestic animals were important at the site and were likely reared using a low-cost free-range system of animal husbandry. This interpretation does not discount the fact that horticultural foods were likely grown and eaten at Teouma, but rather suggests that these plants were not heavily relied upon during the earliest settlement phase in Vanuatu's prehistory.

It is known that Lapita populations brought with them a number of exotic domestic plants and animals as part of a ‘transported landscape’ that was necessary for the establishment of new settlements, but these early colonizers also had to adapt to increasingly sparse conditions as they moved eastward across the Pacific Ocean [7], [175], [176]. The ecology of each specific island, the type of domestic plant and animal species brought by the colonists and later arrivals, the time necessary to establish each crop type and the overall size of the settlement would all have been important factors affecting the success of horticulture and animal husbandry at each site. Depending on the type and number of cultivars brought to Efate initially and through trade or return voyaging after settlement, the establishment and production of imported plant foods (root crops and arboricultural species) would have taken time before yielding enough food to adequately provide for a fledgling community and their domestic animals. The stable isotope evidence may actually be the first direct evidence of a “low-cost free-range system” of animal husbandry as has been posited by Clark et al. [157: 9].

The lack of a significant positive correlation between carbon and nitrogen isotope ratios for the overall sample in addition to the range of values observed for the Teouma humans is very different from the dietary patterns observed at sites later in prehistory where horticulture was heavily relied on, thus supporting the interpretation that these people were a truly colonizing group in Vanuatu. The finding of sex-related dietary variation has also added to the linguistic data by illuminating possible social-cultural factors affecting diet during the heretofore-enigmatic Lapita period of human colonization.

## Supporting Information

Table S1 **Demographic data, bone collagen δ^13^C, δ^15^N and δ^34^S values, collagen quality indicators and laboratory specifics for the humans from Teouma.**
(DOCX)

Table S2 **Bone collagen δ^13^C, δ^15^N and δ^34^S values, temporal period and collagen quality indicators for the prehistoric fauna from Teouma.**
(DOCX)

Table S3 **Modern plant and animal δ^13^C, δ^15^N and δ^34^S values and area of collection in Vanuatu.**
(DOCX)
