# Supplementary material for: Lapita Diet in Remote Oceania: New Stable Isotope Evidence from the 3000-Year-Old Teouma Site, Efate Island, Vanuatu
Source: PLoS One. 2014 Mar 5;9(3):e90376. doi: 10.1371/journal.pone.0090376 (PMC3944017; doi:10.1371/journal.pone.0090376)
Supplement: Table S1 — Demographic data, bone collagen δ13C, δ15N and δ34S values, collagen quality indicators and laboratory specifics for the humans from Teouma. (DOCX) [file pone.0090376.s001.docx]

Table S1. Demographic data, bone collagen δ^13^C, δ^15^N and δ^34^S values, collagen quality indicators and laboratory specifics for the humans from Teouma.

| Burial | Bone sampled | Age^a^ | Sex ^b^ | %C | δ^13^C (‰) | %N | δ^15^N (‰) | C:N | %S | δ^34^S (‰)^c^ | C:S | N:S | Prepared^d^ | Laboratory^e^ |
| --- | --- | --- | --- | --- | --- | --- | --- | --- | --- | --- | --- | --- | --- | --- |
| T1 | Tibia shaft | OA | M | 35.2 | -15.0 | 12.2 | 11.8 | 3.4 | ***0.32*** | ***8.8*** | ***295.8*** | ***87.9*** | U. Otago | Iso-Trace |
| T2a | Rib | OA | M | 50.5 | -15.6 | 17.3 | 12.9 | 3.4 | 0.26 | 10.6 | 518.9 | 152.4 | U. Otago | Iso-Trace |
| T3 | Rib | MA | F | 24.1 | -14.1 | 9.5 | 10.6 | 3.0 |  |  |  |  | U. Otago | Iso-Trace |
| T4 | Rib | MA | M | 24.4 | -17.0 | 9.6 | 13.4 | 3.0 |  |  |  |  | U. Otago | Iso-Trace |
| T5a | Fibula shaft | MA | F | 40.3 | -14.9 | 14.1 | 11.5 | 3.3 | 0.28 | 9.6 | 389.5 | 116.8 | U. Otago | Iso-Trace |
| T6 | Tibia shaft | MA | M | 46.5 | -15.5 | 16.2 | 13.1 | 3.3 | 0.23 | 10.2 | 528.6 | 158.1 | U. Otago | Iso-Trace |
| T7 | Fibula shaft | YA | M | 34.1 | -14.3 | 12.2 | 12.1 | 3.3 | 0.22 | 11.3 | 404.3 | 124.0 | U. Otago | Iso-Trace |
| T8 | Femur shaft | OA | M | 36.5 | -14.5 | 12.7 | 11.8 | 3.4 | 0.26 | 12.1 | 367.4 | 109.6 | U. Otago | Iso-Trace |
| T9 | Scapula | UA | F | 16.4 | -14.2 | 6.6 | 12.1 | 2.9 | ***0.31*** | ***11.4*** | ***142.2*** | ***49.0*** | U. Otago | Iso-Trace |
| T10E | Femur shaft | MA | M | 42.7 | -15.6 | 14.1 | 12.7 | 3.5 | ***0.35*** | ***8.7*** | ***323*** | ***91.7*** | U. Otago | Iso-Trace |
| T11 | Tibia shaft | UA | M | 31.3 | -14.9 | 10.5 | 11.0 | 3.5 |  |  |  |  | U. Otago | Iso-Trace |
| T12 | Tibia shaft | OA | F | 27.0 | -16.8 | 10.3 | 11.3 | 3.1 |  |  |  |  | U. Otago | Iso-Trace |
| T14a | Scapula | OA | M | 20.5 | -16.7 | 7.9 | 12.8 | 3.0 | ***0.25*** | ***12.4*** | ***215.1*** | ***71.1*** | U. Otago | Iso-Trace |
| T15 | Lumbar vertebrae | YA | F | 43.2 | -15.8 | 14.7 | 12.5 | 3.4 | 0.23 | 10.7 | 495.7 | 145.0 | U. Otago | Iso-Trace |
| T16 | Fibula shaft | OA | F | 41.7 | -16.2 | 14.0 | 10.6 | 3.5 | 0.26 | 11 | 426.1 | 122.6 | U. Otago | Iso-Trace |
| T18 | Tibia shaft | YA | M | 40.1 | -15.2 | 13.1 | 11.5 | 3.6 | 0.23 | 11.7 | 457.4 | 128.1 | U. Otago | Iso-Trace |
| T19 | Fibula shaft | MA | M | 41.9 | -16.1 | 14.7 | 12.0 | 3.3 | 0.24 | 10.7 | 471.1 | 141.7 | U. Otago | Iso-Trace |
| T20 | Femur shaft | YA | M | 39.8 | -16.2 | 13.1 | 16.1 | 3.5 | 0.22 | 13.6 | 488.9 | 137.9 | U. Otago | Iso-Trace |
| T23 | Sacrum | YA | M | 9.2 | -15.0 | 3.6 | 13.1 | 3.0 | ***0.11*** | ***10.6*** | ***223.8*** | ***75.1*** | U. Otago | Iso-Trace |
| T25 | Tibia shaft | YA | M | 35.4 | -16.4 | 12.1 | 14.3 | 3.4 | 0.2 | 13.5 | 463.7 | 135.8 | U. Otago | Iso-Trace |
| T27 | Long bone shaft | MA | F | 23.6 | -18.6 | 9.1 | 11.0 | 3.0 | ***0.22*** | ***11.2*** | ***282.6*** | ***93.4*** | U. Otago | Iso-Trace |
| T31 | Scapula | OA | M | 34.6 | -17.4 | 12.3 | 13.0 | 3.3 | 0.27 | 11.3 | 338.6 | 103.2 | U. Otago | Iso-Trace |
| T32 | Long bone shaft | MA | F | 33.5 | -16.7 | 12.2 | 11.0 | 3.2 |  |  |  |  | U. Otago | Iso-Trace |
| T33 | Femur shaft | UA | F | 35.0 | -17.0 | 12.7 | 12.1 | 3.2 |  |  |  |  | U. Otago | Iso-Trace |
| T34 | Humerus shaft | OA | F | 33.5 | -16.1 | 12.1 | 13.0 | 3.2 |  |  |  |  | U. Otago | Iso-Trace |
| T36 | Femur shaft | OA | F | 40.6 | -14.0 | 14.1 | 11.5 | 3.4 | 0.23 | 12.3 | 474.4 | 141.2 | U. Otago | Iso-Trace |
| T37 | Femur shaft | UA | M | 33.7 | -16.0 | 12.4 | 12.5 | 3.2 | 0.22 | 9.5 | 401.2 | 126.5 | U. Otago | Iso-Trace |
| T38 | Tibia shaft | UA | F | 33.5 | -15.8 | 12.8 | 11.6 | 3.1 | ***0.3*** | ***10.6*** | ***294.6*** | ***96.5*** | U. Otago | Iso-Trace |
| T40 | Long bone shaft | YA | F | 35.8 | -15.4 | 13.1 | 12.9 | 3.2 | ***0.1*** | ***10*** | ***954.7*** | ***299.4*** | U. Otago | Iso-Trace |
| T41 | Tibia shaft | YA | F | 33.4 | -15.5 | 12.0 | 10.4 | 3.2 |  |  |  |  | A.M.U. | Iso-Analytical |
| T43 | Tibia shaft | UA | M | 40.1 | -15.9 | 14.2 | 11.5 | 3.3 |  |  |  |  | A.M.U. | Iso-Analytical |
| T44 | Pelvis | OA | M | 35.4 | -18.2 | 12.8 | 13.4 | 3.2 | ***0.3*** | ***10.3*** | ***317.6*** | ***98.4*** | U. Otago | Iso-Trace |
| T47 | Lumbar vertebrae | UA | F | 17.2 | -15.0 | 6.6 | 12.2 | 3.0 |  |  |  |  | U. Otago | Iso-Trace |
| T48 | Ulna shaft | MA | F | 34.1 | -14.4 | 13.0 | 11.4 | 3.1 | ***0.32*** | ***10.6*** | ***285.9*** | ***93.4*** | U. Otago | Iso-Trace |
| T49 | Pelvis | UA | M | 15.6 | -19.3 | 6.2 | 12.2 | 2.9 |  |  |  |  | U. Otago | Iso-Trace |
| T50 | Femur shaft | MA | M | 34.7 | -15.5 | 12.4 | 13.0 | 3.3 |  |  |  |  | A.M.U. | Iso-Analytical |
| T51 | Humerus shaft | UA | M | 23.5 | -15.7 | 8.4 | 13.0 | 3.3 |  |  |  |  | A.M.U. | Iso-Analytical |
| T52 | Tibia shaft | OA | F | 36.8 | -16.2 | 13.3 | 11.5 | 3.2 |  |  |  |  | A.M.U. | Iso-Analytical |
| T53 | Humerus shaft | MA | M | 26.2 | -14.2 | 9.3 | 12.0 | 3.3 |  |  |  |  | A.M.U. | Iso-Analytical |
| T54 | Tibia shaft | OA | M | 41.1 | -13.8 | 14.6 | 12.3 | 3.3 |  |  |  |  | A.M.U. | Iso-Analytical |
| T55 | Fibula shaft | UA | F | 39.7 | -15.9 | 14.2 | 10.8 | 3.2 |  |  |  |  | A.M.U. | Iso-Analytical |
| T56 | Tibia shaft | UA | F | 39.9 | -15.1 | 14.3 | 12.1 | 3.2 |  |  |  |  | A.M.U. | Iso-Analytical |
| T57 | Tibia shaft | MA | F | 38.9 | -16.6 | 13.8 | 11.4 | 3.3 |  |  |  |  | A.M.U. | Iso-Analytical |
| T58 | Fibula shaft | MA | F | 35.7 | -14.5 | 12.7 | 11.4 | 3.3 |  |  |  |  | A.M.U. | Iso-Analytical |
| T59 | Tibia shaft | YA | US | 39.2 | -15.4 | 14.1 | 12.7 | 3.2 |  |  |  |  | A.M.U. | Iso-Analytical |
| T60 | Tibia shaft | UA | F | 38.1 | -13.6 | 13.6 | 11.9 | 3.3 |  |  |  |  | A.M.U. | Iso-Analytical |
| ***T63*** | ***Tibia shaft*** | ***UA*** | ***F*** | ***23.1*** | ***-15.6*** | ***9.8*** | ***6.9*** | ***2.7*** |  |  |  |  | A.M.U. | *Iso-Analytical* |
| ***T64*** | ***Tibia shaft*** | ***YA*** | ***M*** | ***36.3*** | ***-16.4*** | ***15.3*** | ***8.0*** | ***2.8*** |  |  |  |  | A.M.U. | *Iso-Analytical* |
| T65 | Tibia shaft | UA | F | 39.6 | -15.4 | 14.7 | 10.7 | 3.1 |  |  |  |  | A.M.U. | Iso-Analytical |
| T66 | Femur shaft | YA | F | 33.5 | -15.4 | 12.4 | 11.8 | 3.1 |  |  |  |  | A.M.U. | Iso-Analytical |
| T67 | Tibia shaft | MA | F | 38.3 | -14.9 | 14.2 | 11.7 | 3.1 |  |  |  |  | A.M.U. | Iso-Analytical |

^a^ YA = Young adult (20-34 years), MA = Mid adult (35-49 years), OA = Old adult (50 + years), and UA = Adult with unknown age.

^b^ M = Male, F = Female, and US = Unknown Sex.

^c^ Sulfur stable isotope analysis was conducted by EA-IRMS (Europa elemental analyser and mass spectrometer) at Iso-Analytical (Cheshire, UK). Internal standards IAEA-SO-5 (δ^34^S = 0.50 ‰) and IA-R027 (δ^34^S = 16.30 ‰) were run in sets of six alongside the samples for quality control. Analytical precision was calculated from duplicate measurements of the samples and nine repeated measurements of the barium sulfate control IA-R036 (δ^34^S = 20.74 ‰).

^d^ Collagen was extracted at either the University of Otago (Dunedin, NZ) or Aix-Marseille University (Aix-en-Provence, France) using methods described in the text.

^e^ Carbon and nitrogen stable isotope analysis was conducted by EA-IRMS at one of two labs. One lab was Iso-Analytical (Cheshire, UK), which used an Europa elemental analyser and Europa 20-20 mass spectrometer. The internal standards IA-R005 (δ^13^C = -26.03 ‰) and IA-R006 (δ^13^C = -11.64 ‰) for carbon and IA-R045 (δ^15^N = -4.71 ‰) and IA-R046 (δ^15^N = 22.04 ‰) for nitrogen were analysed in sets of eight alongside the samples for quality control. Analytical precision was calculated from duplicate measurements of the samples and eighteen repeated measurements of the bovine liver control NIST-1577B (δ^13^C = -21.60 ‰ and δ^15^N = 7.65 ‰). The other lab was Iso-Trace (Dunedin, NZ), which used a Roboprep CN elemental analyser and Finnigan MAT 252 mass spectrometer. Internal standards NBS22 (δ^13^C = -30.03 ‰) and ANU Sucrose (δ^13^C = -10.8 ‰) for carbon and IAEA N1 (δ^15^N = 0.4 ‰) and IAEA N2 (δ^15^N = 20.3 ‰) for nitrogen were analysed alongside the samples. Analytical precision was calculated from duplicate measurements of the samples and three repeated measurements of the control EDTA (δ^13^C = -38.3 ‰ and δ^15^N = -0.9 ‰).

Bold and italicized samples did not reach the collagen quality criteria outlined in the text.
