# Supplementary material for: Lapita Diet in Remote Oceania: New Stable Isotope Evidence from the 3000-Year-Old Teouma Site, Efate Island, Vanuatu
Source: PLoS One. 2014 Mar 5;9(3):e90376. doi: 10.1371/journal.pone.0090376 (PMC3944017; doi:10.1371/journal.pone.0090376)
Supplement: Table S2 — Bone collagen δ13C, δ15N and δ34S values, temporal period and collagen quality indicators for the prehistoric fauna from Teouma. (DOCX) [file pone.0090376.s002.docx]

Table S2. Bone collagen δ^13^C, δ^15^N and δ^34^S values, temporal period and collagen quality indicators for the prehistoric fauna from Teouma.

| Lab ID | Animal | Scientific name | Element | Layer^a^ | %C | δ^13^C (‰) | %N | δ^15^N (‰) | C:N | %S | δ^34^S (‰) | C:S | N:S | Ref^bcd^ |
| --- | --- | --- | --- | --- | --- | --- | --- | --- | --- | --- | --- | --- | --- | --- |
| TF1 | Chicken | *Gallus gallus* | Tibiotarsus | Lapita | 43.0 | -20.8 | 14.7 | 11.3 | 3.4 | 0.20 | 13.0 | 573.4 | 168.0 | 1 |
| TF2 | Chicken | *Gallus gallus* | Tarsometatarsus | Lapita | 43.4 | -20.5 | 15.1 | 13.1 | 3.4 | 0.20 | 12.3 | 578.2 | 172.3 | 1 |
| TF3 | Chicken | *Gallus gallus* | Tarsometatarsus | Lapita | 44.6 | -20.1 | 15.2 | 10.7 | 3.4 | 0.20 | 12.4 | 594.7 | 173.4 | 1 |
| TF4 | Chicken | *Gallus gallus* | Tarsometatarsus | Lapita | 44.6 | -19.6 | 15.5 | 10.6 | 3.4 | 0.22 | 10.9 | 540.8 | 161.1 | 1 |
| TF5 | Chicken | *Gallus gallus* | Humerus | Lapita | 39.1 | -17.8 | 13.5 | 10.0 | 3.4 | 0.23 | 12.2 | 453.5 | 134.5 | 1 |
| VUTTEO003 | Chicken | *Gallus gallus* | Tarsometatarsus | Post-Lapita | 39.7 | -20.5 | 13.5 | 12.4 | 3.4 |  |  |  |  | 2 |
| VUTTEO006 | Chicken | *Gallus gallus* | Humerus | Lapita | 33.0 | -18.2 | 11.4 | 9.8 | 3.4 |  |  |  |  | 2 |
| TF7 | Pig | *Sus scrofa* | Thoracic vert. | Post-Lapita | 36.0 | -19.3 | 12.4 | 11.6 | 3.4 | 0.19 | 11.9 | 504.8 | 149.2 | 1 |
| TF8 | Pig | *Sus scrofa* | Proximal phalange | Dist. | 42.9 | -20.5 | 15.0 | 9.3 | 3.3 | 0.19 | 10.7 | 602.7 | 180.1 | 1 |
| TF9 | Pig | *Sus scrofa* | Tibia | Post-Lapita | 42.7 | -18.7 | 14.8 | 9.3 | 3.4 | 0.19 | 10.9 | 599.9 | 178.2 | 1 |
| TF10 | Pig | *Sus scrofa* | Thoracic vert. | Post-Lapita | 45.4 | -19.9 | 16.0 | 10.4 | 3.3 | 0.18 | 9.8 | 672.1 | 203.4 | 1 |
| TF11 | Pig | *Sus scrofa* | Lumbar vert. | Post-Lapita | 43.1 | -20.2 | 14.9 | 10.0 | 3.4 | 0.19 | 10.8 | 604.6 | 179.4 | 1 |
| TF12 | Pig | *Sus scrofa* | Rib | Lapita | 43.2 | -19.2 | 15.0 | 10.0 | 3.4 | 0.18 | 11.6 | 640.4 | 191.0 | 1 |
| TF13 | Pig | *Sus scrofa* | Humerus | Lapita | 42.1 | -19.5 | 14.8 | 11.5 | 3.3 | 0.20 | 11.4 | 561.3 | 168.8 | 1 |
| TF14 | Pig | *Sus scrofa* | Thoracic vert. | Lapita | 40.4 | -20.7 | 14.3 | 11.6 | 3.3 | 0.19 | 11.7 | 567.3 | 171.7 | 1 |
| TF15 | Pig | *Sus scrofa* | Humerus | Lapita | 43.2 | -20.1 | 14.9 | 7.8 | 3.4 | 0.20 | 9.7 | 575.9 | 169.9 | 1 |
| TF16 | Pig | *Sus scrofa* | Astragalus | Lapita | 39.9 | -19.6 | 13.9 | 8.6 | 3.3 | 0.18 | 10.2 | 591.4 | 177.1 | 1 |
| TF17 | Pig | *Sus scrofa* | Radius | Lapita | 43.0 | -19.4 | 15.0 | 8.6 | 3.4 | 0.20 | 10.1 | 573.9 | 171.2 | 1 |
| TF18 | Pig | *Sus scrofa* | Mandible | Lapita | 43.3 | -18.3 | 15.3 | 9.1 | 3.3 | 0.19 | 11.0 | 607.8 | 184.1 | 1 |
| TF19 | Pig | *Sus scrofa* | Lumbar vert. | Lapita | 45.0 | -19.7 | 15.8 | 8.8 | 3.3 | 0.20 | 11.2 | 600.3 | 181.0 | 1 |
| TF20 | Pig | *Sus scrofa* | 5th metacarpal | Lapita | 41.2 | -18.7 | 14.7 | 9.0 | 3.3 | 0.19 | 10.0 | 578.1 | 176.5 | 1 |
| TF21 | Pig | *Sus scrofa* | 3rd metacarpal | Lapita | 43.4 | -18.3 | 15.4 | 8.8 | 3.3 | 0.17 | 10.3 | 680.9 | 206.7 | 1 |
| TF22 | Pig | *Sus scrofa* | Lumbar vert. | Lapita | 43.3 | -20.6 | 15.2 | 9.5 | 3.3 | 0.17 | 11.4 | 678.6 | 204.2 | 1 |
| TF23 | Pig | *Sus scrofa* | Pelvis | Lapita | 42.0 | -20.1 | 14.7 | 9.8 | 3.3 | 0.17 | 11.2 | 659.1 | 197.2 | 1 |
| TF24 | Pig | *Sus scrofa* | Rib | Lapita | 41.4 | -20.3 | 14.4 | 9.5 | 3.3 | 0.13 | 12.9 | 849.0 | 253.7 | 1 |
| TF03V | Pig | *Sus scrofa* | Humerus | Post-Lapita | 29.8 | -20.1 | 10.7 | 9.8 | 3.3 |  |  |  |  | 3 |
| TF04V | Pig | *Sus scrofa* | Femur | Post-Lapita | 35.6 | -18.9 | 12.8 | 8.3 | 3.3 |  |  |  |  | 3 |
| TF26 | Tortoise | *?Meiolania damelipi* | Femur | Lapita | 41.0 | -23.6 | 13.5 | 7.4 | 3.5 |  |  |  |  | 1 |
| TF27 | Tortoise | *?Meiolania damelipi* | Fibula | Lapita | 44.1 | -23.7 | 15.2 | 2.4 | 3.4 | 0.15 | 10.4 | 784.0 | 230.9 | 1 |
| TF28 | Tortoise | *?Meiolania damelipi* | Humerus | Late Lapita | 44.7 | -23.8 | 15.1 | 3.1 | 3.5 |  |  |  |  | 1 |
| TF29 | Tortoise | *?Meiolania damelipi* | Femur | Dist. | 43.0 | -22.8 | 14.4 | 3.4 | 3.5 |  |  |  |  | 1 |
| TF30 | Tortoise | *?Meiolania damelipi* | Femur | Late Lapita | 43.6 | -23.9 | 14.5 | 1.5 | 3.5 | 0.17 | 10.4 | 683.3 | 195.2 | 1 |
| ***TF31*** | ***Tortoise*** | ***?Meiolania damelipi*** | ***Humerus*** | ***Late Lapita*** | ***36.2*** | ***-23.9*** | ***10.7*** | ***3.1*** | ***3.9*** |  |  |  |  | 1 |
| TF32 | Tortoise | *?Meiolania damelipi* | Humerus | Dist. | 43.1 | -24.1 | 14.3 | 2.6 | 3.5 | 0.14 | 11.3 | 821.5 | 233.6 | 1 |
| TF33 | Tortoise | *?Meiolania damelipi* | Femur | Dist. | 43.0 | -22.4 | 14.7 | 8.8 | 3.4 | 0.16 | 12.0 | 717.1 | 210.3 | 1 |
| TF35 | Tortoise | *?Meiolania damelipi* | Pelvis | Lapita | 42.5 | -23.6 | 14.1 | 5.7 | 3.5 |  |  |  |  | 1 |
| TF36 | Tortoise | *?Meiolania damelipi* | Pelvis | Lapita | 44.7 | -23.2 | 15.7 | 5.6 | 3.3 |  |  |  |  | 1 |
| TF38 | Tortoise | *?Meiolania damelipi* | Pelvis | Lapita | 41.7 | -23.0 | 14.4 | 8.2 | 3.4 |  |  |  |  | 1 |
| TF39 | Tortoise | *?Meiolania damelipi* | Pelvis | Lapita | 45.7 | -22.8 | 15.8 | 7.5 | 3.4 |  |  |  |  | 1 |
| TF42 | Tortoise | *?Meiolania damelipi* | Pelvis | Lapita | 40.8 | -23.3 | 13.7 | 8.9 | 3.5 |  |  |  |  | 1 |
| TF44 | Tortoise | *?Meiolania damelipi* | Pelvis | Lapita | 43.7 | -23.4 | 14.7 | 8.4 | 3.5 |  |  |  |  | 1 |
| TF46 | Tortoise | *?Meiolania damelipi* | Pectoral | Lapita | 41.4 | -23.9 | 13.7 | 0.8 | 3.5 |  |  |  |  | 1 |
| TF47 | Tortoise | *?Meiolania damelipi* | Pectoral | Lapita | 42.2 | -23.7 | 13.8 | 2.4 | 3.6 |  |  |  |  | 1 |
| Wk25601 | Tortoise | *?Meiolania damelipi* | ? | Late Lapita | 43.4 | -23.2 | 15.0 | 3.0 | 3.4 |  |  |  |  | 4 |
| WK25602 | Tortoise | *?Meiolania damelipi* | ? | Late Lapita | 42.2 | -22.8 | 15.0 | 3.3 | 3.3 |  |  |  |  | 4 |
| TF49 | Marine turtle | *Chelonia mydas?* | Ulna | Lapita | 44.2 | -10.8 | 14.5 | 13.2 | 3.6 |  |  |  |  | 1 |
| TF51 | Marine turtle | *Chelonia mydas?* | Phalange | Lapita | 39.6 | -11.6 | 13.3 | 13.0 | 3.5 |  |  |  |  | 1 |
| ***TF52*** | ***Marine turtle*** | ***Chelonia mydas?*** | ***Femur*** | ***Lapita*** | ***43.4*** | ***-18.2*** | ***10.0*** | ***14.9*** | ***5.1*** |  |  |  |  | 1 |
| TF54 | Fruit bat | Pteropodidae | Radius | Lapita | 43.8 | -19.5 | 15.5 | 5.2 | 3.3 |  |  |  |  | 1 |
| TF55 | Fruit bat | Pteropodidae | Humerus | Lapita | 44.6 | -19.8 | 15.5 | 3.4 | 3.4 |  |  |  |  | 1 |
| TF56 | Fruit bat | Pteropodidae | Humerus | Lapita | 46.9 | -20.1 | 16.4 | 6.0 | 3.3 |  |  |  |  | 1 |
| TF57 | Fruit bat | Pteropodidae | Humerus | Lapita | 44.5 | -20.2 | 15.8 | 3.8 | 3.3 |  |  |  |  | 1 |
| TF58 | Fruit bat | Pteropodidae | Humerus | Lapita | 42.9 | -20.2 | 15.3 | 3.1 | 3.3 |  |  |  |  | 1 |
| TF59 | Fruit bat | Pteropodidae | Humerus | Lapita | 43.4 | -20.1 | 15.4 | 3.5 | 3.3 |  |  |  |  | 1 |
| TF60 | Fruit bat | Pteropodidae | Humerus | Lapita | 44.4 | -19.0 | 15.8 | 3.8 | 3.3 |  |  |  |  | 1 |
| TF61 | Fruit bat | Pteropodidae | Humerus | Lapita | 43.2 | -20.2 | 15.5 | 6.9 | 3.3 |  |  |  |  | 1 |
| TF62 | Fruit bat | Pteropodidae | Humerus | Post-Lapita | 43.5 | -20.2 | 15.2 | 6.1 | 3.3 |  |  |  |  | 1 |
| TF63 | Fruit bat | Pteropodidae | Humerus | Post-Lapita | 42.4 | -19.8 | 14.8 | 3.7 | 3.4 |  |  |  |  | 1 |
| TF64 | Fruit bat | Pteropodidae | Radius | Post-Lapita | 43.8 | -20.3 | 15.6 | 6.1 | 3.3 |  |  |  |  | 1 |
| TF65 | Fruit bat | Pteropodidae | Humerus | Post-Lapita | 41.7 | -19.6 | 14.4 | 4.8 | 3.4 |  |  |  |  | 1 |
| TF66 | Fruit bat | Pteropodidae | Humerus | Post-Lapita | 39.5 | -19.6 | 14.0 | 6.2 | 3.3 |  |  |  |  | 1 |
| TF67 | Fruit bat | Pteropodidae | Humerus | Post-Lapita | 41.8 | -19.6 | 14.5 | 8.1 | 3.4 |  |  |  |  | 1 |
| TF68 | Fruit bat | Pteropodidae | Humerus | Post-Lapita | 41.9 | -19.6 | 14.5 | 8.4 | 3.4 |  |  |  |  | 1 |
| TF69 | Fruit bat | Pteropodidae | Humerus | Post-Lapita | 43.2 | -20.0 | 15.1 | 3.0 | 3.3 |  |  |  |  | 1 |
| TF70 | Fruit bat | Pteropodidae | Humerus | Post-Lapita | 40.3 | -19.7 | 14.4 | 6.8 | 3.3 |  |  |  |  | 1 |
| TF71 | Fruit bat | Pteropodidae | Humerus | Late Lapita | 42.4 | -19.8 | 15.0 | 3.7 | 3.3 |  |  |  |  | 1 |
| TF72 | Fruit bat | Pteropodidae | Humerus | Late Lapita | 43.9 | -20.3 | 15.6 | 7.4 | 3.3 |  |  |  |  | 1 |
| TF73 | Fruit bat | Pteropodidae | Humerus | Late Lapita | 42.9 | -19.8 | 15.2 | 5.5 | 3.3 |  |  |  |  | 1 |
| TF01V | Fruit bat | Pteropodidae | Humerus | Post-Lapita | 39.4 | -19.5 | 14.5 | 5.0 | 3.2 |  |  |  |  | 3 |
| TF02V | Fruit bat | Pteropodidae | Metatarsal | Post-Lapita | 34.7 | -19.0 | 12.6 | 4.5 | 3.2 |  |  |  |  | 3 |
| TF05V | Rat | *Rattus exulans* | Long Bone | Post-Lapita | 37.8 | -18.9 | 13.2 | 8.7 | 3.4 |  |  |  |  | 3 |
| TF06V | Rat | *Rattus exulans* | Corticol bone | Post-Lapita | 33.4 | -19.4 | 12.2 | 10.4 | 3.2 |  |  |  |  | 3 |
| TF07 | Reef fish | Scaridae | Dentary | Post-Lapita | 28.6 | -10.9 | 10.2 | 8.1 | 3.3 |  |  |  |  | 3 |

^a^ Dist=disturbed deposits, likely mixing of post-Lapita and Lapita layers.

^b^ 1. This study; 2. Storey et al. (119); 3. Valentin et al. (32); 4. White et al. (102).

^c^ In this study carbon and nitrogen stable isotope analysis was conducted by EA-IRMS at Iso-Analytical (Cheshire, UK), using an Europa elemental analyser and Europa 20-20 mass spectrometer. The internal standards IA-R005 (δ^13^C = -26.03 ‰) and IA-R006 (δ^13^C = -11.64 ‰) for carbon and IA-R045 (δ^15^N = -4.71 ‰) and IA-R046 (δ^15^N = 22.04 ‰) for nitrogen were analysed in sets of eight alongside the samples for quality control. Analytical precision was calculated from duplicate measurements of the samples and eighteen repeated measurements of the bovine liver control NIST-1577B (δ^13^C = -21.60 ‰ and δ^15^N = 7.65 ‰).

^d^ In this study sulfur stable isotope analysis was conducted by EA-IRMS (Europe elemental analyser and mass spectrometer) at Iso-Analytical (Cheshire, UK). Internal standards IAEA-SO-5 (δ^34^S = 0.50 ‰) and IA-R027 (δ^34^S = 16.30 ‰) were run in sets of six alongside the samples for quality control. Analytical precision was calculated from duplicate measurements of the samples and nine repeated measurements of the barium sulfate control IA-R036 (δ^34^S = 20.74 ‰).

Bold and italicized samples did not reach the collagen quality criteria outlined in the text.
