# Supplementary material for: Lapita Diet in Remote Oceania: New Stable Isotope Evidence from the 3000-Year-Old Teouma Site, Efate Island, Vanuatu
Source: PLoS One. 2014 Mar 5;9(3):e90376. doi: 10.1371/journal.pone.0090376 (PMC3944017; doi:10.1371/journal.pone.0090376)
Supplement: Table S3 — Modern plant and animal δ13C, δ15N and δ34S values and area of collection in Vanuatu. (DOCX) [file pone.0090376.s003.docx]

Table S3. Modern plant and animal δ^13^C, δ^15^N and δ^34^S values and area of collection in Vanuatu.

| Lab ID | Common name^a^ | Scientific name | Village/Region^b^ | Island | %C^c^ | δ^13^C (‰) | %N | δ^15^N (‰) | C:N | %S^d^ | δ^34^S (‰) | C:S | N:S |
| --- | --- | --- | --- | --- | --- | --- | --- | --- | --- | --- | --- | --- | --- |
| OT1 | Kava | *Piper methysticum* | NW Malakula | Malakula | 38.3 | -26.3 | 0.3 | 3.5 | 133.7 |  |  |  |  |
| OT2 | Kava | *Piper methysticum* | NW Malakula | Malakula | 43.5 | -27.0 | 0.4 | 2.2 | 142.8 |  |  |  |  |
| OT3 | Dragon plum | *Dracontomelon vitiense* | Uripiv | Malakula | 40.5 | -27.1 | 1.3 | 2.8 | 35.5 | 0.08 | 9.2 | 1350.3 | 38.0 |
| OT4 | Banana | *Musa* sp. | ML Malakula | Malakula | 38.9 | -24.8 | 0.4 | 1.6 | 120.0 |  |  |  |  |
| OT5 | Banana | *Musa* sp. | Uripiv | Malakula | 40.1 | -23.9 | 0.4 | 5.2 | 135.2 |  |  |  |  |
| OT6 | Banana | *Musa* sp. | Uripiv | Malakula | 39.4 | -24.3 | 0.3 | 4.9 | 158.5 |  |  |  |  |
| OT7 | Banana | *Musa* sp. | Uripiv | Malakula | 40.8 | -26.3 | 1.0 | 5.5 | 49.5 |  |  |  |  |
| OT8 | Banana | *Musa* sp. | Uripiv | Malakula | 41.0 | -31.6 | 1.9 | 6.0 | 25.4 |  |  |  |  |
| OT9 | Yam | *Dioscorea* spp*.* | Uripiv | Malakula | 40.5 | -27.6 | 0.5 | 1.0 | 97.3 |  |  |  |  |
| OT10 | Yam | *Dioscorea* spp*.* | Uripiv | Malakula | 40.3 | -25.7 | 0.3 | 2.4 | 137.6 |  |  |  |  |
| OT11 | Yam | *Dioscorea* spp*.* | Uripiv | Malakula | 40.3 | -26.6 | 1.1 | 4.2 | 43.6 |  |  |  |  |
| OT12 | Yam | *Dioscorea* spp*.* | Uripiv | Malakula | 38.9 | -29.1 | 1.1 | 5.6 | 43.4 | 0.07 | 3.7 | 1483.0 | 34.3 |
| OT13 | Yam | *Dioscorea* spp*.* | Uripiv | Malakula | 40.8 | -27.5 | 1.6 | 0.5 | 30.6 |  |  |  |  |
| OT14 | Taro | *Colocasia esculenta* | ML Malakula | Malakula | 39.7 | -27.4 | 0.6 | 4.9 | 81.5 |  |  |  |  |
| OT15 | Taro | *Colocasia esculenta* | ML Malakula | Malakula | 38.9 | -25.9 | 0.5 | 4.1 | 96.5 |  |  |  |  |
| OT16 | Taro | *Colocasia esculenta* | Uripiv | Malakula | 39.2 | -25.3 | 0.6 | 2.2 | 78.9 |  |  |  |  |
| OT17 | Taro | *Colocasia esculenta* | Uripiv | Malakula | 39.3 | -24.7 | 0.7 | 4.6 | 70.9 |  |  |  |  |
| OT18 | Taro | *Colocasia esculenta* | Uripiv | Malakula | 39.7 | -25.1 | 0.7 | 3.7 | 69.6 |  |  |  |  |
| OT19 | Coconut | *Cocos nucifera* | ML Malakula | Malakula | 52.9 | -25.6 | 5.1 | 5.2 | 12.1 |  |  |  |  |
| OT20 | Coconut | *Cocos nucifera* | ML Malakula | Malakula | 50.7 | -26.3 | 5.4 | 4.0 | 10.9 |  |  |  |  |
| OT21 | Coconut | *Cocos nucifera* | Uripiv | Malakula | 41.9 | -23.7 | 5.1 | 5.8 | 9.5 |  |  |  |  |
| OT22 | Coconut | *Cocos nucifera* | Uripiv | Malakula | 45.0 | -24.5 | 3.5 | 3.8 | 15.0 |  |  |  |  |
| OT23 | Coconut | *Cocos nucifera* | Uripiv | Malakula | 42.7 | -24.6 | 1.5 | 1.3 | 34.4 | 0.13 | 4.8 | 876.7 | 25.5 |
| OT24 | Coconut | *Cocos nucifera* | Uripiv | Malakula | 41.5 | -24.3 | 3.1 | 5.8 | 15.5 |  |  |  |  |
| OT25 | Sugarcane | *Saccharum officinarum* | Uripiv | Malakula | 41.3 | -12.5 | 0.4 | 3.4 | 119.3 |  |  |  |  |
| OT26 | Sugarcane | *Saccharum officinarum* | Uripiv | Malakula | 42.4 | -12.7 | 0.5 | 5.0 | 91.3 |  |  |  |  |
| OT27 | Sugarcane | *Saccharum officinarum* | Uripiv | Malakula | 40.5 | -12.5 | 0.7 | 3.2 | 72.7 |  |  |  |  |
| OT28 | Canarium nut | *Canarium* spp*.* | Uripiv | Malakula | 40.2 | -24.8 | 10.7 | 2.7 | 4.4 |  |  |  |  |
| OT29 | Canarium nut | *Canarium* spp. | Uripiv | Malakula | 42.1 | -27.1 | 8.6 | 8.6 | 5.7 |  |  |  |  |
| OT30 | Canarium nut | *Canarium* spp. | Uripiv | Malakula | 43.5 | -25.9 | 8.6 | 5.3 | 5.9 |  |  |  |  |
| OT31 | Canarium nut | *Canarium* spp*.* | Uripiv | Malakula | 35.4 | -26.2 | 9.4 | 2.3 | 4.4 |  |  |  |  |
| OT32 | Canarium nut | *Canarium* spp. | Wala ML | Malakula | 42.4 | -27.9 | 10.2 | 7.5 | 4.8 |  |  |  |  |
| OT33 | Tropical almond | *Terminalia catappa* | Uripiv | Malakula | 44.5 | -24.4 | 11.1 | 1.4 | 4.7 |  |  |  |  |
| OT34 | Cutnut | *Barringtonia* spp*.* | Uripiv | Malakula | 44.9 | -22.6 | 2.9 | 7.7 | 17.8 | 0.20 | 11.8 | 599.2 | 33.6 |
| OT35 | Cutnut | *Barringtonia* spp*.* | Uripiv | Malakula | 41.1 | -22.6 | 3.8 | 6.0 | 12.5 | 0.40 | 8.2 | 274.1 | 21.9 |
| OT36 | Tahitian chestnut | *Inocarpus fagifer* | Uripiv | Malakula | 42.8 | -28.3 | 5.6 | 3.1 | 8.9 |  |  |  |  |
| OT37 | Seagrass | *Thalassia hemprichii* | Uripiv | Malakula | 38.4 | -8.2 | 3.3 | 1.0 | 13.4 | 0.33 | 14.0 | 310.5 | 23.1 |
| OT38 | Seagrass | *Thalassia hemprichii* | Uri | Malakula | 34.6 | -5.2 | 1.9 | 0.9 | 20.9 |  |  |  |  |
| OT39 | Seagrass | *Syringodium isoetifolium* | Uri | Malakula | 37.6 | -14.6 | 1.2 | 1.8 | 35.8 |  |  |  |  |
| OT40 | Sea pursalene | *Sesuvium portulacastrum* | Uripiv | Malakula | 38.2 | -26.7 | 3.0 | 0.9 | 14.7 |  |  |  |  |
| OT41 | Marine turtle bone | *Chelonia mydas or Eretmochelys imbricata* | Uripiv | Malakula | 45.3 | -9.7 | 17.0 | 9.2 | 3.1 | 0.23 | 13.6 | 524.9 | 169.0 |
| OT42 | Sardine bone | *Sardinella fijiense* | Uripiv | Malakula | 38.5 | -14.3 | 14.1 | 5.8 | 3.2 |  |  |  |  |
| OT43 | Sardine bone | *Sardinella fijiense* | Uripiv | Malakula | 44.5 | -14.3 | 16.7 | 5.8 | 3.1 | 0.46 | 17.2 | 258.0 | 82.8 |
| OT44 | Parrotfish bone | Scaridae | Uripiv | Malakula | 42.3 | -12.4 | 15.9 | 7.6 | 3.1 | 0.39 | 17.6 | 289.4 | 93.0 |
| OT45 | Parrotfish bone | Scaridae | Uripiv | Malakula | 46.0 | -7.0 | 17.6 | 3.2 | 3.1 |  |  |  |  |
| OT46 | Longfish bone | Sphyraenidae | Uripiv | Malakula | 44.2 | -12.3 | 16.0 | 6.2 | 3.2 |  |  |  |  |
| OT47 | Longfish bone | Sphyraenidae | Uripiv | Malakula | 40.7 | -12.2 | 15.0 | 5.8 | 3.2 |  |  |  |  |
| OT48 | Tuna bone | Scombridae | Uripiv | Malakula | 43.5 | -13.0 | 15.4 | 7.5 | 3.3 |  |  |  |  |
| OT49 | Tuna bone | Scombridae | Uripiv | Malakula | 47.8 | -12.9 | 18.0 | 7.1 | 3.1 | 0.46 | 18.2 | 277.2 | 89.4 |
| OT52 | Fruit bat bone | Pteropodidae | Eratap | Efate | 46.2 | -21.7 | 16.2 | 7.1 | 3.3 | 0.25 | 11.0 | 492.3 | 148.3 |
| OT53 | Chalky buttercup* | *Anodontia philippiana* | Uri | Malakula | 41.6 | -28.9 | 10.0 | -9.5 | 4.9 | 0.97 | -21.5 | 114.4 | 23.5 |
| OT54 | Gastropod | *Thais tuberosa* | Uri | Malakula | 42.5 | -8.8 | 12.7 | 5.8 | 3.9 |  |  |  |  |
| OT55 | Mangrove gastropod* | [Cerithiidae](http://en.wikipedia.org/wiki/Cerithiidae) | Uri | Malakula | 36.7 | -24.9 | 6.8 | 0.9 | 6.3 | 1.22 | 4.0 | 80.2 | 12.7 |
| OT56 | Small sea snail | Neritidae | Uri | Malakula | 41.4 | -7.0 | 11.7 | 3.3 | 4.1 |  |  |  |  |
| OT57 | Bivalve | Psammobiidae | Uripiv | Malakula | 39.9 | -5.7 | 8.5 | 1.2 | 5.5 |  |  |  |  |
| OT58 | Bivalve | *Arcopagia (Pinguitellina) robusta* | Uripiv | Malakula | 44.8 | -5.6 | 13.8 | 4.7 | 3.8 |  |  |  |  |
| OT59 | Mangrove gastrapod* | [Cerithiidae](http://en.wikipedia.org/wiki/Cerithiidae) | Uripiv | Malakula | 42.3 | -10.2 | 12.4 | 4.6 | 4.0 |  |  |  |  |
| OT60 | Lettered Cone | *Conus litteratus* | Uripiv | Malakula | 44.6 | -15.6 | 13.4 | 6.2 | 3.9 |  |  |  |  |
| OT61 | Tiger Cowrie | *Cypraea tigris* | Uripiv | Malakula | 45.4 | -9.2 | 14.4 | 7.5 | 3.7 |  |  |  |  |
| OT62 | Chiton | Acanthochitonidae | Uripiv | Malakula | 41.9 | -1.1 | 13.1 | 2.5 | 3.7 | 0.81 | 14.9 | 137.8 | 36.9 |
| OT63 | Thick-shelled clam | Veneridae | Eratap | Efate | 42.4 | -4.8 | 13.3 | 6.1 | 3.7 |  |  |  |  |
| OT64 | Scallop | Pectinidae | Eratap | Efate | 46.6 | -15.0 | 14.5 | 6.4 | 3.7 |  |  |  |  |
| OT65 | Halfround cardita | *Beguina semiorbiculata* | Eratap | Efate | 46.9 | -14.5 | 13.7 | 7.4 | 4.0 |  |  |  |  |
| OT66 | Pacific oyster | *Crassostrea gigas* | Eratap | Efate | 39.1 | -16.0 | 11.7 | 5.0 | 3.9 |  |  |  |  |
| OT67 | Lettered Cone | *Conus litteratus* | Eratap | Efate | 42.2 | -9.2 | 12.3 | 7.6 | 4.0 |  |  |  |  |
| OT68 | Tiger Cowrie | *Cypraea tigris* | Eratap | Efate | 40.3 | -14.0 | 10.6 | 7.5 | 4.4 |  |  |  |  |
| OT69 | Tiger Cowrie | *Cypraea tigris* | Eratap | Efate | 37.9 | -15.0 | 9.7 | 6.9 | 4.6 |  |  |  |  |
| OT70 | Mangrove gastropod* | [Cerithiidae](http://en.wikipedia.org/wiki/Cerithiidae) | Eratap | Efate | 42.1 | -24.5 | 12.5 | 2.6 | 3.9 |  |  |  |  |
| OT71 | Island cabbage | *Abelmoschus manihot* | Eratap | Efate | 38.2 | -29.4 | 4.4 | 9.8 | 10.3 | 0.55 | 12.4 | 185.2 | 18.1 |
| OT72 | Island cabbage | *Abelmoschus manihot* | Eratap | Efate | 38.2 | -29.1 | 5.3 | 5.1 | 8.5 |  |  |  |  |
| OT73 | Taro | *Colocasia esculenta* | Teouma | Efate | 40.1 | -25.6 | 0.8 | 4.7 | 61.4 |  |  |  |  |
| OT74 | Taro | *Colocasia esculenta* | Teouma | Efate | 39.5 | -26.6 | 0.8 | 4.3 | 60.8 |  |  |  |  |
| OT75 | Taro | *Colocasia esculenta* | Teouma | Efate | 40.1 | -29.9 | 0.4 | 2.3 | 116.5 |  |  |  |  |
| OT76 | Taro | *Colocasia esculenta* | Teouma | Efate | 38.7 | -27.7 | 0.4 | 0.7 | 112.2 |  |  |  |  |
| OT77 | Taro | *Colocasia esculenta* | Teouma | Efate | 40.2 | -28.2 | 0.3 | 1.6 | 157.2 |  |  |  |  |
| OT78 | Yam | *Dioscorea* spp*.* | Teouma | Efate | 39.4 | -29.9 | 1.3 | 2.8 | 35.0 |  |  |  |  |
| OT79 | Yam | *Dioscorea* spp*.* | Teouma | Efate | 39.9 | -25.6 | 0.6 | 2.4 | 79.5 |  |  |  |  |
| OT80 | Yam | *Dioscorea* spp*.* | Teouma | Efate | 39.7 | -30.1 | 1.1 | 3.0 | 40.9 | 0.07 | 10.1 | 1510.5 | 36.9 |
| OT81 | Cutnut | *Barringtonia* spp*.* | Teouma | Efate | 39.6 | -25.0 | 6.8 | 3.3 | 6.8 |  |  |  |  |
| OT82 | Cutnut | *Barringtonia* spp*.* | Teouma | Efate | 41.2 | -23.4 | 3.3 | 6.4 | 14.5 | 0.30 | 15.7 | 365.9 | 25.1 |
| OT83 | Canarium nut | *Canarium* spp*.* | Epule | Efate | 41.6 | -28.3 | 6.8 | 4.4 | 7.1 |  |  |  |  |
| OT84 | Canarium nut | *Canarium* spp*.* | Epau | Efate | 40.1 | -26.9 | 9.3 | 7.5 | 5.1 |  |  |  |  |
| OT85 | Banana | *Musa* sp*.* | Teouma | Efate | 40.4 | -25.7 | 0.5 | 3.0 | 88.4 |  |  |  |  |
| OT86 | Banana | *Musa* sp*.* | Teouma | Efate | 40.1 | -24.5 | 0.5 | 2.1 | 95.8 |  |  |  |  |
| OT87 | Banana | *Musa* sp*.* | Teouma | Efate | 40.3 | -24.7 | 0.6 | 1.9 | 82.5 |  |  |  |  |
| OT88 | Banana | *Musa* sp*.* | Teouma | Efate | 39.7 | -29.5 | 1.3 | 3.4 | 36.1 |  |  |  |  |
| OT89 | Banana | *Musa* sp*.* | Teouma | Efate | 40.8 | -30.1 | 1.0 | 3.4 | 48.0 |  |  |  |  |
| OT90 | Banana | *Musa* sp*.* | Teouma | Efate | 38.7 | -29.1 | 0.5 | 3.7 | 85.2 |  |  |  |  |
| OT91 | Coconut | *Cocos nucifera* | Epule | Efate | 39.2 | -24.4 | 0.7 | 2.6 | 64.5 |  |  |  |  |
| OT92 | Coconut | *Cocos nucifera* | Epule | Efate | 41.9 | -23.9 | 1.0 | 4.5 | 48.8 |  |  |  |  |
| OT93 | Coconut | *Cocos nucifera* | Eratap | Efate | 44.2 | -25.3 | 3.9 | 4.8 | 13.1 |  |  |  |  |
| OT94 | Coconut | *Cocos nucifera* | Eratap | Efate | 52.7 | -26.1 | 2.4 | 4.6 | 25.9 |  |  |  |  |
| OT95 | Coconut | *Cocos nucifera* | Teouma | Efate | 43.5 | -22.6 | 3.7 | 4.8 | 13.7 |  |  |  |  |
| OT96 | Coconut | *Cocos nucifera* | Teouma | Efate | 38.2 | -23.7 | 3.6 | 3.6 | 12.3 |  |  |  |  |
| OT97 | Breadfruit | *Artocarpus altilis* | Paonangisu | Efate | 40.5 | -23.7 | 0.6 | 3.4 | 85.0 |  |  |  |  |
| OT98 | Breadfruit | *Artocarpus altilis* | Paonangisu | Efate | 39.6 | -23.8 | 0.6 | 2.9 | 76.1 |  |  |  |  |
| OT99 | Crab* | *Scylla serrata* | ML Malakula | Malakula | 46.1 | -24.1 | 13.8 | 2.9 | 3.9 |  |  |  |  |
| OT100 | Crab* | *Scylla serrata* | ML Malakula | Malakula | 47.7 | -24.1 | 15.1 | 2.1 | 3.7 | 0.96 | 9.4 | 132.4 | 35.9 |

^a^ * Denotes mangrove species

^b^ Northwest Malakula (NW Malakula), mainland Malakula (ML Malakula) Wala mainland (Wala ML)

^c^ Samples were prepared at the University of Otago (Dunedin, New Zealand) using methods described in the text. In this study carbon and nitrogen stable isotope analysis was conducted by EA-IRMS at Iso-Analytical (Cheshire, UK), using an Europa elemental analyser and Europa 20-20 mass spectrometer. The internal standards IA-R005 (δ^13^C = -26.03 ‰) and IA-R006 (δ^13^C = -11.64 ‰) for carbon and IA-R045 (δ^15^N = -4.71 ‰) and IA-R046 (δ^15^N = 22.04 ‰) for nitrogen were analysed in sets of eight alongside the samples for quality control. Analytical precision was calculated from duplicate measurements of the samples and eighteen repeated measurements of the bovine liver control NIST-1577B (δ^13^C = -21.60 ‰ and δ^15^N = 7.65 ‰).

^d^ Sulfur stable isotope analysis was conducted by EA-IRMS (Europe elemental analyser and mass spectrometer) at Iso-Analytical (Cheshire, UK). Internal standards IAEA-SO-5 (δ^34^S = 0.50 ‰) and IA-R027 (δ^34^S = 16.30 ‰) were run in sets of six alongside the samples for quality control. Analytical precision was calculated from duplicate measurements of the samples and nine repeated measurements of the barium sulfate control IA-R036 (δ^34^S = 20.74 ‰).
